# Evidence of Heteroepitaxy and Solid Solutions in Lattice Matched Ternary Covalent Organic Framework Systems

**DOI:** 10.1021/jacs.5c02502

**Published:** 2025-06-02

**Authors:** Alena Winter, Juliane Lange, Farzad Hamdi, Panagiotis L. Kastritis, Frederik Haase

**Affiliations:** † Institute of Chemistry, Faculty of Natural Sciences II, 9176 Martin-Luther-University Halle-Wittenberg, von-Danckelmann-Platz 4, 06120 Halle (Saale), Germany; ‡ Department of Integrative Structural Biochemistry, Faculty of Natural Sciences 1—Biosciences Martin-Luther University, Weinbergweg 22, 06120 Halle (Saale), Germany; § Interdisciplinary Research Center HALOmem, Charles Tanford Protein Center, Martin Luther University Halle-Wittenberg, Kurt-Mothes-Straße 3a, 06120 Halle (Saale), Germany; ∥ Biozentrum, Martin Luther University Halle-Wittenberg, Weinbergweg 22, 06120 Halle (Saale), Germany; ⊥ Institute of Chemical Biology, National Hellenic Research Foundation, Leof. Vasileos Konstantinou 48, Athina, 116 35 Athens, Greece

## Abstract

Covalent organic frameworks (COFs) are crystalline, porous materials with the possibility for broad applications, but their structural diversity remains constrained by simple net topologies, limiting functional versatility. To address this challenge, we developed a strategy for incorporating linkers with normally mismatched geometries, exemplified by a [4-c + 2-c + 3-c] system incorporating pentagonal motifs for 2D tiling. Central to this approach is the derivation of a length ratio parameter, α, which provides a quantitative guide for evaluating the compatibility of linkers in ternary systems. Investigating a model system with close to ideal α, we demonstrate that precise size matching enables the formation of localized solid solutions and heteroepitaxial interfaces, as seen by transmission electron microscopy. These findings showcase a pathway for expanding the structural and functional complexity of COFs, opening new avenues for tailored material design.

## Graphical abstract

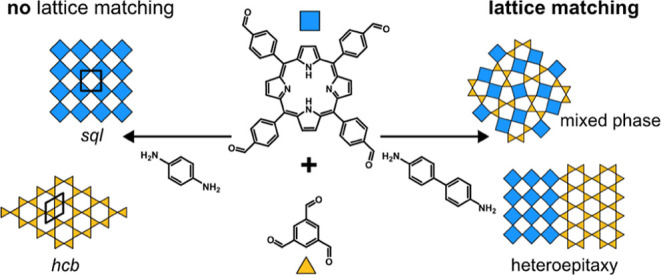

## Introduction

Covalent organic frameworks (COFs) are materials constructed from organic building blocks that form ordered, covalently connected structures in two or three dimensions. The majority of COFs have only two-dimensional (2D) covalent connectivity due to the scarcity of suitable three-dimensional (3D) building blocks.^1^ Most of 2D COF chemistry is constrained to a few predominant nets in which no matching of the building block sizes is necessary and the linkers do not need to be distorted to form periodic structures: [3 + 2] (*hcb*), [3 + 3] (*hcb*), [4 + 2] (*sql*, *kgm*), [4 + 4] (*sql*), [6 + 2] (*hxl*), and [6 + 3] (*kgd*).^1^ (The shortened notation [*m* + *n*] describes the connectivity of all of the building blocks used to construct the COF, including linear 2-c sections, where *m* and *n* represents the number of connections (*m*-c, *n*-c) of the component building blocks.) Efforts to expand this range have included using distorted building blocks or substoichiometric structures, as seen in [5 + 2] (*cem*)^2^ and [4 + 2 + 3] (*bex*)^3^ systems, respectively. More complex structures from normally incompatible linker geometries can be achieved by matching linker sizes, such as the [4 + 4] COF composed of tetrafunctional porphyrin and tetrafunctional quarterphenyl-based linkers recently reported.^4–6^

Expanding linker size matching to [4 + 3] or [4 + 2 + 3] systems opens the door to a much broader array of potential nets. The Reticular Chemistry Structure Resource database lists 65 2D nets that combine three-connected (3-c) and four-connected (4-c) nodes.^7^ As most of these are dual nets of square–triangle tilings, an endless number of periodic, aperiodic, or disordered tilings of the 2D plane is theoretically possible. The square–triangle tilings can theoretically fill the plane without gaps or overlaps, allowing for their dual nets to produce stoichiometric compounds ranging from well-defined simple nets and aperiodic structures to solid solutions.^8^

Solid solutions in COFs form when different linkers share the same lattice, which have been explored by mixing linkers of varying sizes. A continuous variation in linker composition then leads to gradual changes in unit cell size, as evidenced by shifting peaks by powder X-ray diffraction (PXRD). This phenomenon demonstrates the exceptional flexibility of COF backbones to incorporate different linker sizes.^9,10^ Instead of incorporating all linkers into a mixed phase, the size matching of linkers can also lead to heteroepitaxy, where aligned crystalline layers grow on a substrate crystal with a well-defined interface. Heteroepitaxy in COFs has only sparsely been investigated on isostructural COFs with the same lattice parameters.^11–13^

In this work, we started from the hypothesis that we can enable the formation of structures based on dual nets of square–triangle tilings if the sizes of the linkers are precisely matched to each other. This matching would allow for a range of different structural responses: (1) the combination of linkers into highly mixed stoichiometric 2D nets such as *mcm* and *usm* or even all the way to dodecagonal quasiperiodic tilings,^14^ (2) stabilization of the interface between clusters with a local *hcb* or *sql* structure such as in *krm-d* and *krx-d*, or (3) in the extreme case, stabilize grain boundaries between crystallites of *hcb* and *sql* structures (Figure 1A).

**Figure 1:**
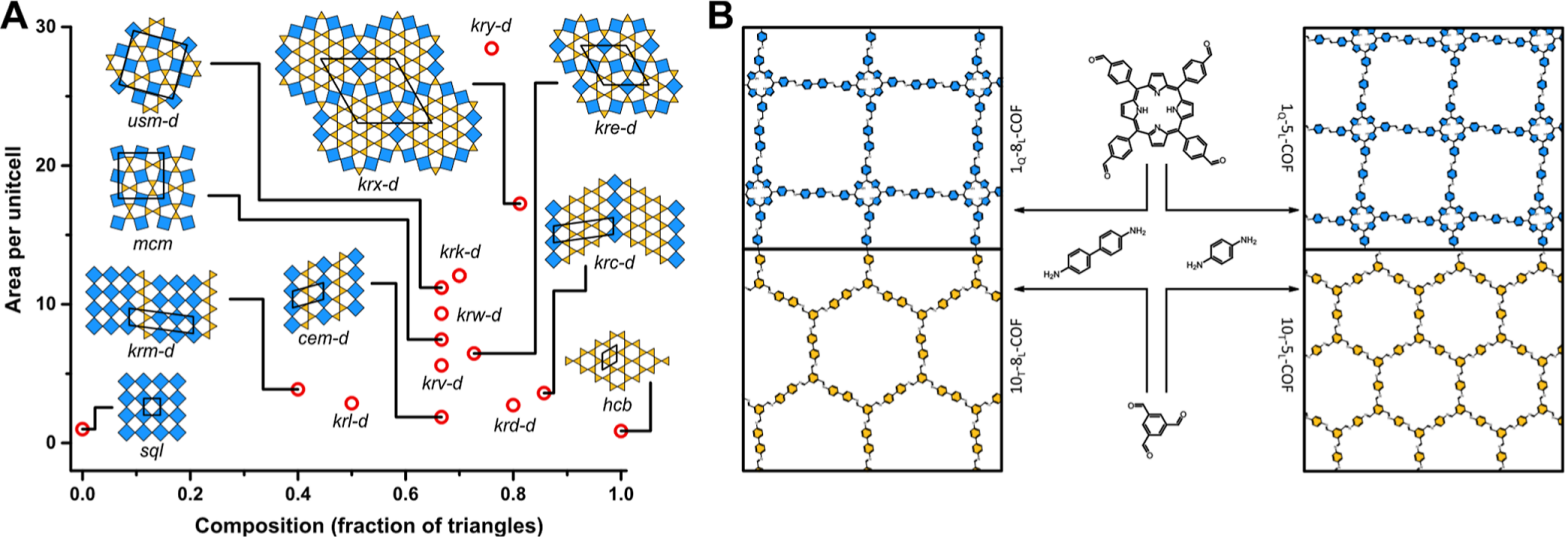
(A) Phase diagram of a selection of possible periodic square–triangle tilings, where mixed phases display pentagonal pores and (B) schematic of the pure phase COFs synthesized here.

Based on this hypothesis, we simulated a range of 2D COFs to find appropriately size-matched linker combinations in a [4 + 2 + 3] linker system. We investigated the most promising candidate in detail, which showed a good match in linker size. We observed that in mixed linker synthesis conditions, pure COF, heteroepitaxial growth, and apparent linker mixing occur. These results indicate that the linker matching strategy might open the door to structurally complex 2D COFs.

## Results and Discussion

### Calculations

Since COFs are formed by self-assembly under reversible conditions, the formation of mixed nets requires that these structures be energetically comparable to, or more stable than, the individual pure phases. On the molecular level this means that the linker molecules need to be able to form the mixed structures without distortion, as this would incur an energetic penalty with respect to the pure *sql* or *hcb* phases. In all mixed structures, pentagonal structural motifs are present that we termed *shield* and the *kite* (Figures 1A and 2). The *kite* and *shield* pentagons can be constructed by connecting the centers of three equilateral triangles and two squares tiled around a node with the sequences 3.3.4.3.4 and 3.3.3.4.4, respectively (Figure 2).^8,16^

**Figure 2:**
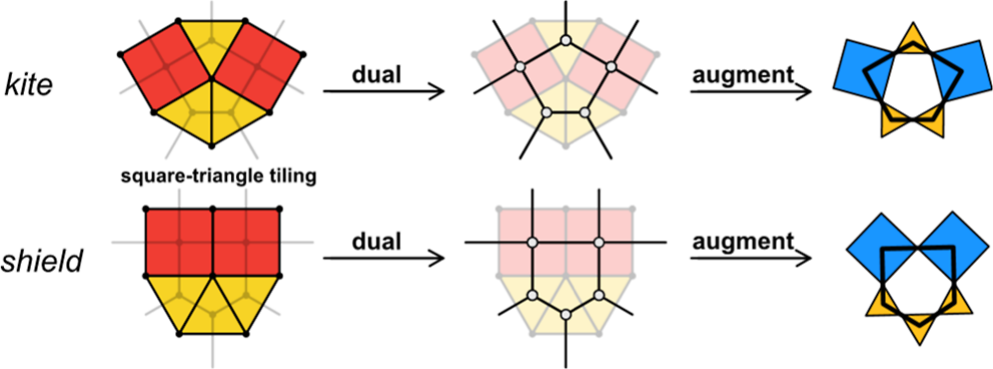
Illustration of the relationship between the square–triangle tilings and their dual nets.

The square–triangle tilings in their highest symmetry embedding lead to nodes with 120° and 90° between edges, which can be easily realized in molecular building blocks. While the net could also be realized with a lower symmetry, it would be difficult to translate to synthesizable molecular building blocks. In order to translate this to a material, we chose the imine-linked COFs as a platform due to their great variety of commercially available linkers and their favorable crystallization behavior and stability.^1,17^ We chose to construct our imine COFs from pure amine and pure aldehyde building blocks, instead of mixed amine–aldehyde building blocks, which have been reported before.^18,19^ As the pentagonal pores have an uneven number of nodes around the pore, using only an amine-based node and an aldehyde-based node is not possible. In our design of the COFs, we therefore also include a 2-connected (2-c) building block leading to the fully alternating [4 + 2 + 3] system.

In order to satisfy the fixed linker angles, the linker sizes need to be precisely matched to form the pentagonal structural motif without distortion. Considering all three building blocks, both types of pentagons can be formed if the following equation of linker sizes is satisfied (Figure 3B, full proof in the Supporting Information):

1 $$\alpha_{0}:=\frac{2t+l}{q+l+t}=\sqrt{3}-1\approx 0.732$$

where α_0_ is the ideal linker size ratio and *q*, *l*, and *t* are the lengths of the 4-c, 2-c, and 3-c linkers, respectively. This value α is comparable to values determined for binary pentagonal pore COFs in previous works that only consider the ratio of the overall side length.^4–6^ The key difference is that our approach is applicable to ternary COFs, which does not limit the nets to the previously reported *sql* topologies but allows theoretically the construction of all nets based on square–triangle tilings.

**Figure 3:**
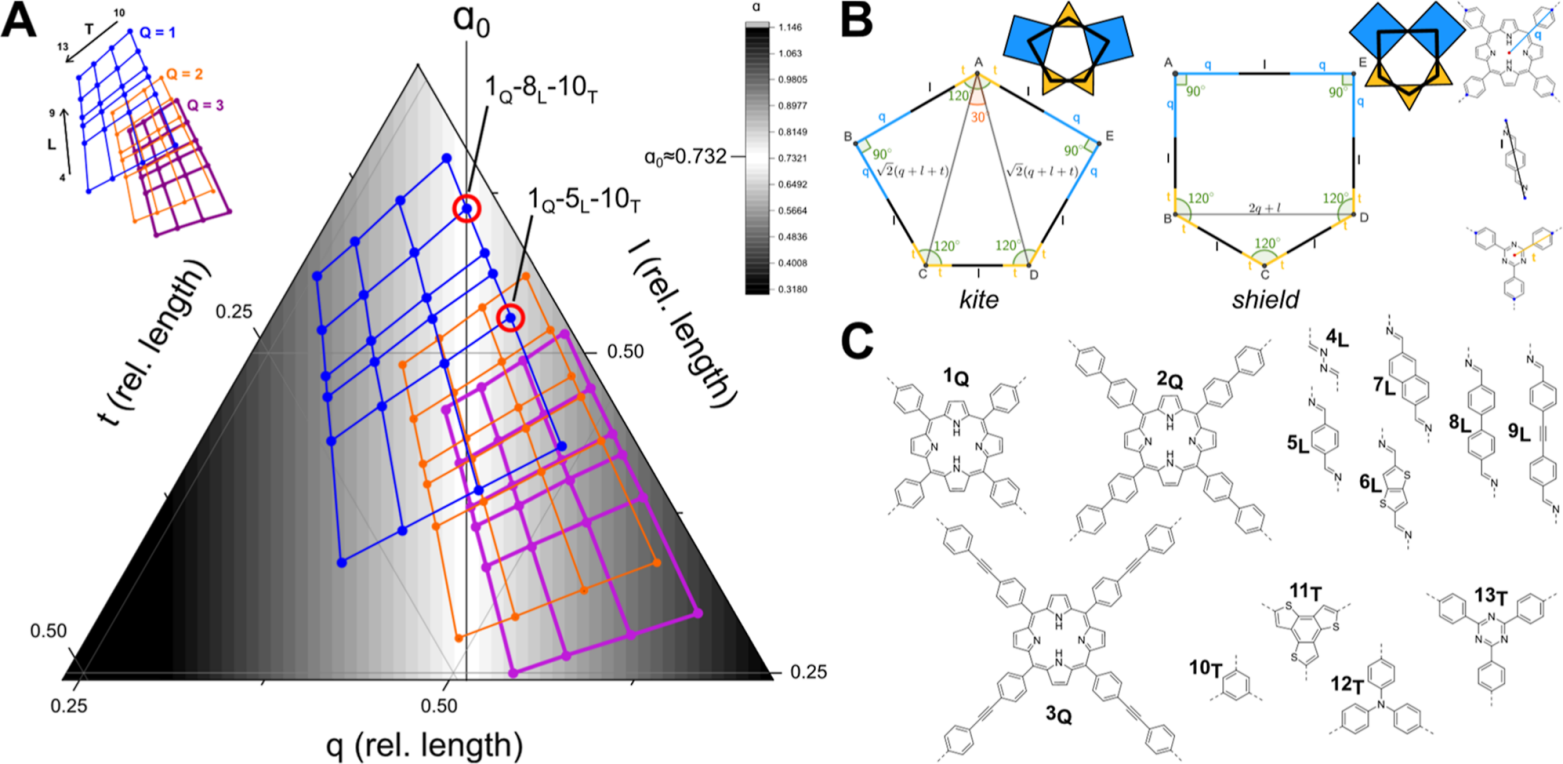
(A) Ternary diagram of the relative lengths of *q*, *l*, and *t* in relation to α_0_ (normalized to *q* + *l* + *t* = 1 for each 3-tuple). The background shading indicates the deviation of α from α_0_ with white being the closest to α_0_. Each dot describes an element consisting of a 4-c, 2-c, and 3-c building block. Each grid highlights a set of combinations for a fixed 4-c building block. The identity of each point can be determined from the legend. (B) Depiction of the two pentagons with the linker lengths marked. (C) Molecular structures of the fragments used for the calculations. The orientation of imines does not affect the distance calculations and was not varied.

Based on this relationship between the lengths *q*, *l*, *t*, and α, we explored a range of common organic building block cores, which can be used for the formation of imine COFs. Linker lengths were approximated by simple geometric lengths based on fully 2D structures, with linker core lengths calculated from individual bond lengths and angles and force field geometry optimized structures. We calculated α for all permutations of the three building block combinations (Figure 3C: square building blocks 1_Q_–3_Q_, linear building blocks 4_L_–9_L_, and triangular building blocks, 10_T_–13_T_). This resulted in a set of linker combinations, which was ranked for the smallest deviation from α_0_ (Table S2) or can be visually mapped onto a ternary diagram (Figure 3A). One of the best candidates, with an α value closely matching the ideal α_0_ value, was benzene (3-c) + biphenyl (2-c) + *meso* tetraphenyl porphyrin (4-c) connected by imine bridges.

### COF Synthesis

Next, we decided to evaluate our computational prediction by synthesizing the 1_Q_-8_L_-10_T_-COF, which had a close to ideal predicted α value (α = 0.732, deviation from α_0_< 1%) and the 1_Q_-5_L_-10_T_-COF (see Supporting Information) with a α value that deviates from the ideal value (α = 0.672, deviation from α_0_ ∼ 9%) to validate our approach.

The computational design specifies only the geometry and size of the backbone and does not consider other factors that might influence the synthesis of the COFs. Moving to experimental verification of these predictions, the first consideration is choosing one of the two possible orientations of the imine bond: (1) triformyl benzene (**10-CHO**) + benzidine (**8-NH**_**2**_) + *meso* tetra­(formylphenyl)­porphyrin (**1-CHO**) and (2) triamino benzene (**10-NH**_**2**_) + diformyl biphenyl (**8-CHO**) + *meso* tetra­(aminophenyl)­porphyrin (**1-NH**_**2**_) (Figure S4). The latter had the advantage of a more accessible porphyrin linker, but we failed to synthesize any triamino benzene-based COFs even when just using the triamino benzene and diformyl biphenyl linkers.

We therefore focused on the system 1 based on three well-established COF linkers that each individually have been used in a range of other COFs.^20–23^ We started the synthesis of the two component COFs based on triformyl benzene (10_T_, see Figure 3C) + benzidine (8_L_) and *meso* tetra­(formylphenyl)­porphyrin (1_Q_) + benzidine (8_L_), which resulted in the 10_T_-8_L_-COF and 1_Q_-8_L_-COF, respectively, the latter of which has been reported before.^15^ Both COFs were first optimized for crystallinity individually (Figure S5). In order to create reliable mixed linker synthesis, we next optimized synthesis conditions that lead to crystalline materials for both COFs in the same reaction conditions. This succeeded with a mixture of *o*-dichlorobenzene/*n*-butanol (1:1) and 3 M acetic acid.

In typical COF synthesis conditions, the COF formation starts without fully dissolving the precursors, which leads to heterogeneous nucleation of the COF on the precursor crystals. This is due to either a lack of time or solubility of the precursor. In two component COFs, this is not an issue since only one reaction product can form. In the ternary linker system, local concentration differences due to undissolved linkers or due to heterogeneous nucleation might lead to preferential formation of binary COFs instead of ternary COFs. To favor homogeneous nucleation, we dissolved the aldehydes in *o*-dichlorobenzene at 90 °C before the addition of the benzidine and acetic acid. Subsequently, the COFs were allowed to react at 120 °C for 3 days before workup by methanol Soxhlet extraction and supercritical CO_2_ drying.

### PXRD Analysis

To verify the successful formation of the COFs, we performed PXRD. The crystallinity of the pure COFs was confirmed by the presence of multiple sharp reflections. These also matched with the calculated PXRD pattern of structures (Figure 4A, see also the Supporting Information) constructed and geometry optimized in Materials Studio. Pawley refinement provided lattice parameters for each of the pure phases. The *a* and *b* parameters of the 8_L_-10_T_-COF were refined with a *hcb* lattice and *P*6 symmetry, which yielded cell parameters of *a* = *b* = 28.95 Å, *c* = 3.53 Å, α = β = 90°, and γ = 120°. The 1_Q_-8_L_-COF was modeled with a *sql* lattice, which clearly exhibited a symmetry reduction to *P*1 (Figure 4A),^24^ the lattice parameters were refined to *a* = *b* = 29.88 Å, *c* = 4.689 Å, with angles α = 114.16°, β = 89.32°, and γ = 96.17°. In both cases, the *c* parameter was kept fixed due to the presence of only *hk*0 reflections in the experimental PXRD data.

**Figure 4:**
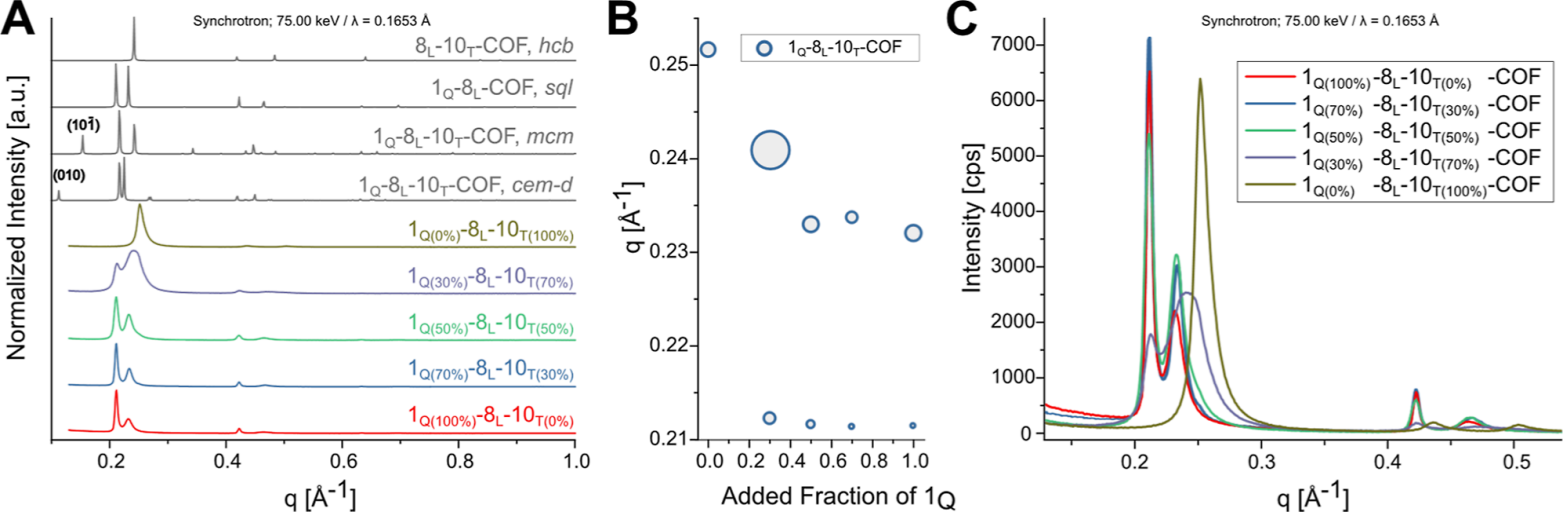
Simulated PXRD of the *hcb*, *mcm*, *cem-d*, and *sql* structures for the 1_Q_-8_L_-10_T_-COF system compared with high-resolution powder X-ray diffraction (HR-PXRD) data (λ = 0.1653 Å) (A). Peak positions of the first and second reflex compared to the composition of the ternary COF (B); the size of the points reflect the FWHM. HR-PXRD data of mixed ternary COF (C).

As the value of α can also be expressed as a function of the lattice parameters (derivation in the Supporting Information), this allows the experimental values obtained from Pawley refinement to be used:

2 $$\alpha_{\exp}:=\frac{2a_{hcb}}{\sqrt{3}a_{sql}+a_{hcb}}$$

where α_exp_ is calculated from the lattice parameter *a*_*hcb*_ of the hexagonal phase and *a*_*sql*_ of the square lattice phase.

The experimental α value of 0.718 for the 1_Q_-10_T_-8_L_ system deviates more than anticipated from the predicted value of 0.732. The lattice parameter *a* of the (*hcb*) 10_T_-8_L_-COF is much smaller in the experimentally obtained value than in the simulated value (1.06 Å). In contrast, the refined lattice parameter *a* of (*sql*) 1_Q_-8_L_-COF deviates only marginally from the simulated *a* (0.3 Å). Since the lattice parameter *a* deviates more strongly in the *hcb*-COF than the *sql*-COF, the experimental α deviates from the predicted α. Nevertheless, the 1_Q_-10_T_-8_L_ system is close to the ideal value of α_0_ and therefore we next investigated the synthesis of COFs based on all three linkers in different stoichiometries.

Previous studies have continuously substituted a shorter 2-c with a longer 2-c linker in a COF with a 3-c linker.^9,10^ There it was observed that solid solutions were formed from ternary linker mixtures even for different linker lengths. We wanted to investigate the continuous substitution of a 4-c linker with a 3-c linker from binary [4-c + 2-c] COF system toward a [3-c + 2-c] system to search for the gradual behavior of the system to linker mixing where the end points of a mixing series do not have the same topology. The 1_Q_-8_L_-10_T_ mixed COFs were synthesized by substituting the aldehyde in increments from 0% to 100% (1_Q(100%)_-8_L_-10_T(0%)_-COF to 1_Q(0%)_-8_L_-10_T(100%)_-COF). Digestion of COF samples showed consistent incorporation of both 1_Q_ and 10_T_ linkers in the solid obtained after synthesis (Figure S5).

First, to determine if new phases with new topologies were obtained, we measured HR-PXRD and small-angle X-ray diffraction (SAXS) at the synchrotron and compared them to simulated patterns. HR-PXRD of the 1_Q_-8_L_-10_T_-COF with *Q* = 100%, 70%, 50%, 30%, and 0% showed for all increments, crystalline materials with varying degrees of peak width and position, but no significant new peaks for the ternary COFs (*Q* = 70%, 50%, and 30%). We constructed models of *mcm* and *cem-d* COFs as representative mixed-phase structures, which have both small unit cell sizes and only one 4-c and one 3-c linker in the asymmetric unit (Figure 1A). The most intense reflexes for the *mcm* and *cem-d* models of a 1_Q_-8_L_-10_T_-COF show similar *q* values to the *sql* and *hcb* models in the range of *q* = 0.211–0.269 Å^–1^ and are therefore difficult to differentiate even in the high-resolution measurements. However, in the low-angle region, additional and highly characteristic new reflexes are apparent in the calculated PXRD patterns. The 101̅ reflex in *mcm* and 010 reflex in *cem-d* would be expected at 0.154 Å^–1^ and 0.112 Å^–1^, respectively. The high-resolution data show no presence of these low-angle reflections. To verify this, the samples were also measured under SAXS conditions at the synchrotron, which also did not show any evidence of additional peaks (Figure S6).

The absence of these small-angle reflections suggests that long-range-ordered mixed phases such as *mcm* or *cem-d* are not present in the ternary 1_Q_-8_L_-10_T_-COFs. However, several factors make the detection of ordered mixed phases difficult by PXRD. Small domain mixed phase structural features would not be expected to be visible in the PXRD, due to crystallite size broadening. Simulated PXRD patterns of multiple ordered mixed phases (Figures S6 and S8) show that the expected low *q* reflexes are low in intensity, making them difficult to detect even under high-resolution conditions. The predicted positions of low-*q* reflections for various ordered mixed phases differ substantially in reflex position depending on the specific topology. As a result, any distribution of such phases would cause these reflections to be distributed over a wide *q*-range, leading to an increase in background rather than distinct peaks. This stands in contrast to the high-intensity reflections around *q* = 0.2–0.3 Å^–1^, where different structural motifs produce signals in the same *q*-region that constructively overlap to form the broad double reflexes observed in our experimental PXRD data. Third, in the case of solid solution phases, low-*q* reflections are not expected at all. Instead, as confirmed by transmission electron microscopy (TEM) imaging (see below), these regions exhibit diffuse fast Fourier transform (FFT) rings corresponding to the same *q*-range as the PXRD peaks. This is indicative of local short-range order without long-range periodicity, resembling the behavior of structurally disordered or glassy materials.

For the 1_Q(0%)_-8_L_-10_T(100%)_-COF, we observed the most intense peak at 0.252 Å^–1^, which corresponds to the 100 peak of the *hcb*-COF, whereas for the 1_Q(100%)_-8_L_-10_T(0%)_-COF, two peaks at 0.211 and 0.232 Å^–1^ can be detected, corresponding to the 100 and 010 peaks of the *sql*-COF. The 010 peak of the *sql* lattice partially overlaps with the 100 of the *hcb* lattice, which increases the complexity of the analysis. Throughout the mixing series, as the proportion of 1_Q_ decreases, the position of the first peak remains constant, while the second peak shifts to higher *q* values. This trend is apparent in both synchrotron and low-resolution PXRD data. For low-resolution PXRD, finer increments were used (Figure 5). The high-resolution synchrotron data reveal significant broadening of the second peak with decreasing fractions of 1_Q_ (Figure 4B). This effect is lost in the low-resolution PXRD, where no second reflex is detectable for the 1_Q(100%)_-8_L_-COF, due to instrumental line broadening. At the same time, the relative intensity of the first peak decreases with respect to the second peak.

**Figure 5:**
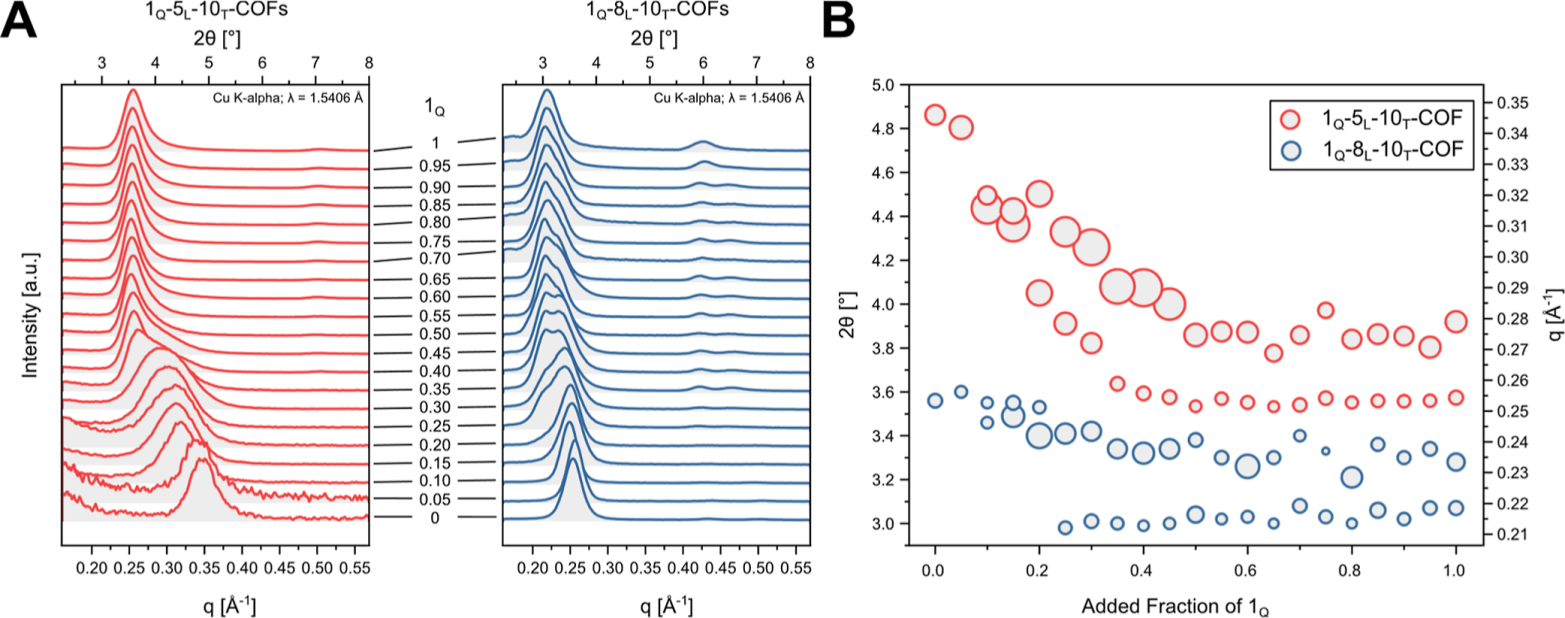
PXRD of fine-grained ternary mixtures of 1_Q_-5_L_-10_T_-COF (Supporting Information) (left) and 1_Q_-8_L_-10_T_-COF (right) (A) and peak fit each point was determined based on the relative FWHM of the peaks to one another at the given content of 1_Q_ (B).

Nonetheless, the low-resolution PXRD, performed in 5% increments, allowed the fine-grained analysis of the peak positions, where a cursory examination showed that for the 1_Q(45%)_-8_L_-10_T(55%)_-COF, both peaks became nearly equal in intensity and remained this way until 1_Q(35%)_-8_L_-10_T(65%)_-COF, followed by a rapid decrease in intensity for the first peak and a shift to higher 2θ values for the second peak (Figure 4A). To quantify this effect, we performed a peak fit of the overlapping peaks with a pseudo-Voigt peak function. The peak position plotted against the added fraction of (Figure 5B) revealed that the peak position of the lower angle peak remains constant for a content of 1_Q_ of 100% to 25%. After that, the peak intensity is too low to obtain a reliable fit. In contrast, the higher angle peak continuously shifts position toward higher angles with decreasing content of 1_Q_. The continuous peak shift and intensity change suggest a continuous variation of the composition in one phase, which is constituent with a formation of a solid solution.^10^

The formation of a 4-c/3-c solid solution should only be possible if the linker sizes are matched to α to allow square–triangle tilings. To verify, we did the same experiments and analysis on the 1_Q_-5_L_-10_T_-COF (Supporting Information, Figure 5). This showed, in contrast, that the low- and high-angle peak position remained constant from 1_Q_ = 100% to 50% and then rapidly shifted together toward higher angles. This suggests a low compatibility evidenced by a smaller region of mixing. In a true two-phase system, a jump in peak position would be expected. However, the low reversibility of COF crystallization might prevent the complete phase separation.

Solid solutions or new mixed phases should also be easy to distinguish in TEM, since it provides close-up insight into the heterogeneity and morphology in real space. As a representative example, we chose to look deeper into the 1_Q(50%)_-8_L_-10_T(50%)_-COF sample.

### Transmission Electron Microscopy

The TEM images revealed a morphologically unusually heterogeneous material with at least four distinct morphologies that were observed throughout. We termed these morphologies “haystack”, “hollow spheres”, “intergrown crystallites”, and “dilute network”. Each morphology showed characteristic lattice fringes at higher magnification. The “haystack morphology” consisted of small, intergrown needle-shaped crystallites with a length of 250–350 nm and thickness of 20 to 40 nm with lattice spacing and a local symmetry, which corresponds to the *sql* structure (Figure S9). The “hollow spheres” ranged in size from 175 to 675 nm with distinct walls of a thickness of 50 to 200 nm, which agglomerate. They exhibit a pure *hcb* phase with a lattice spacing of 2.54 nm (Figure S10). The “dilute network” is composed of 100 and 250 nm sized crystallites, featured poorly defined edges, and largely exhibits a *hcb* phase with some epitaxial growth of square lattices attaching seamlessly to the *hcb* network (Figures 7, S13, and S15). As the fourth phase, the “intergrown crystallites” phase ranges in crystallite sizes from 20 to 275 nm depending on the location, primarily forming an *hcb* network, interdispersed with *sql* and mixed lattice arrangements (Figures S11 and S12). The “haystack” and “hollow spheres” phases show crystallites of pure phase *sql* and *hcb* topology, respectively. Those match in their symmetry and *d*-spacings with the molecular models and PXRD of these materials. The “haystack” 1_Q_-8_L_-COF shows crystallites (Figure S7) with a square lattice with *d*-spacings of 2.98 nm corresponding to the 100 reflection in the PXRD, which matches well with the 2.98 nm observed for the 100 reflection. The value of γ can also be determined from the TEM images, which is ∼96°, which is a close match with the 96.17° obtained from the Pawley refinement. The “dilute network” phase is dominated by larger crystallites of the *hcb*-COF (Figure S8) with a hexagonal lattice and symmetry. It has a *d*-spacing of 2.54 nm, which matches well with the 2.504 nm obtained from Pawley refinement (Figure S13).

In addition to the crystallites, which can be clearly identified as either *sql* or *hcb* (Figures S7 and S8), there are widespread regions in the “intergrown crystallites” phase that display a mixture of features from both *sql* and *hcb*. Here, both structures can be observed as separate crystallites, but also features of both hexagonal and square lattices can be observed within one crystallite/grain (Figure 7). Additionally, fully disordered regions that nevertheless show a ring in the FFT with a *d*-spacing of ∼2.59 nm can be observed (Figures 5A and S19). This lattice parameter is close to the *d*-spacing of the *sql* and *hcb* phase. The *hcb* or *sql* domains can be arbitrarily small to relatively large in these images (Figure 6E). In some crystallites, local structures are reminiscent of idealized mixed phases. For example, four fused pentagons (Figure 6C) are characteristic of a *mcm* structure (Figure 6B). The observed feature dimensions align closely with force field calculations (6.2 × 8.8 nm) and TEM measurements (6.2 × 8.2 nm).

**Figure 6:**
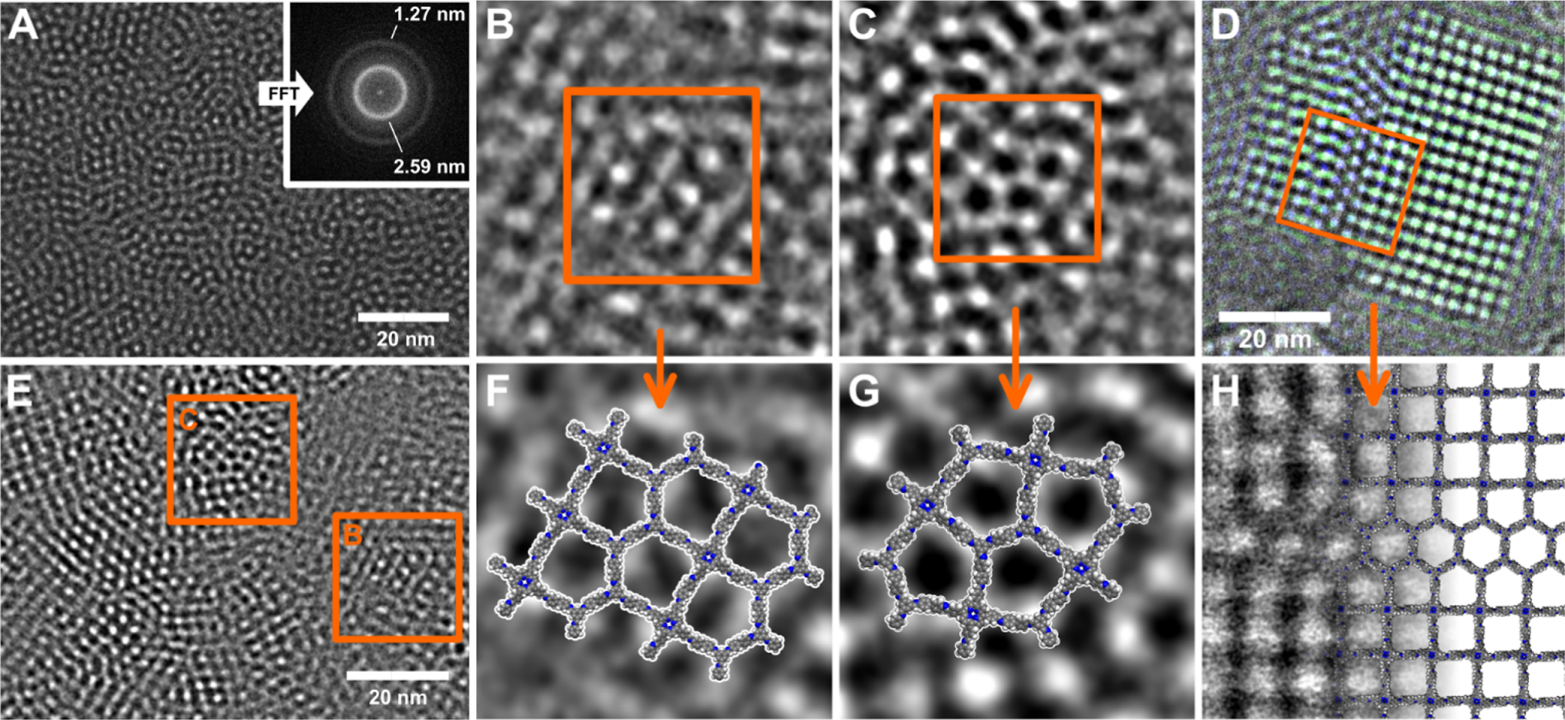
TEM images of solid solutions and mixed phases (A,E) in the “intergrown crystallites” phase of the 1_Q(50%)_-8_L_-10_T(50%)_-COF showing. Four fused pentagons (B,C) similar to the pentagons observed in the mcm structure (F) and alternating spots and stripes (G) that could be attributed to a structural motif also occurring in the *cem-d* structure. Mixed hexagonal and square lattice crystallite (D,H) (B, C, E–F filtered with a bandpass filter).

In the regions of mixed phases, the FFT shows a single broad peak from 2.34 to 3.14 nm (Figure S21A,B), while regions of *sql* and *hcb* crystallites in close proximity tend to give well-resolved sharper peaks in the same region in the FFT (Figure S21C,D). In these structures, there are no clear crystallite or grain boundaries; essentially, features of both *hcb* and *sql* are intermixed at the small length scales, giving a strong indication of solid solutions. Although it is inherently challenging to distinguish overlapping crystallites from intergrown or mixed-phase COFs by their FFT alone, the images generated by overlapping crystallites show striking visual differences from the mixed phase (Figure S17), an effect that could also be attributed to multilayer phase contrast. In order to estimate the fraction of the sample that is composed of solid solution, we used automated acquisition of images over an entire TEM grid. Prior to acquisition, we manually selected regions containing visible sample material to avoid the collection of empty images. This combination allowed for both efficient and unbiased image acquisition of over 6000 images. Next, we excluded images without visible lattice fringes. We then manually analyzed the TEM images and categorized them into containing only pure phases (*sql* or *hcb*), only solid solutions, or both solid solutions and pure phases. The analysis of the 589 useable TEM images resulted in 308 with only solid solutions, 148 with only pure phases, and 133 with both pure phases and solid solutions. This result shows that solid solutions appear as the major components of the sample on the TEM grid.

While the “dilute network” morphology is dominated by larger *hcb* domains, there are additional smaller domains with larger *d*-spacing present. These domains are especially frequent at the *hcb* crystallite edges. Differentiating these phases is possible by their lattice spacing: while the *hcb* phase (8_L_-10_T_-COF) possesses a clear hexagonal symmetry with a periodicity of ∼2.54 nm, the larger *d*-spacing of ∼2.74–2.98 nm matches well with the *sql* phase (1_Q_-8_L_-COF). This allows the differentiation of both phases, even in the absence of the local symmetry information. Curiously, nearly all of the grain boundary angles between the two phases are close to 30° (Figure 7A,B,D and S15), which closely matches the ideal grain boundary angle for heteroepitaxy to occur between a *sql* and *hcb* phase of 30° (Figures 1B and 7F). This crystallite alignment observed in heteroepitaxy also creates a distinct FFT pattern, where the spot corresponding to the 100 reflection of the *sql* lattice is located between the 100 and 11̅0 spots of the *hcb* lattice (Figures 7C,E and S16).

**Figure 7:**
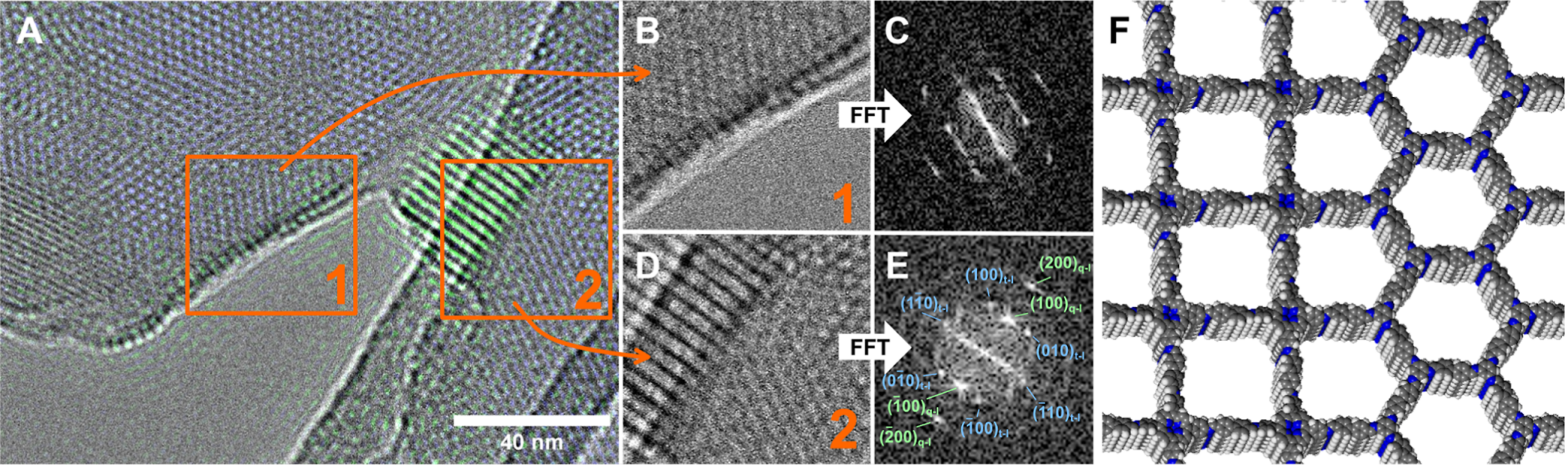
Heteroepitaxial interfaces in the 1_Q_-8_L_-10_T_-COF. (A) TEM image of two different aligned heteroepitaxial interfaces between the *sql* phase and the *hcb* phase. Color overlays are based on the *d*-spacing observed in the corresponding regions: green higher *d*-spacing component (∼2.94 nm) corresponding to the *sql* phase; blue corresponds to the lower *d*-spacing component (∼2.41 nm), which corresponds to the *hcb* phase in this image. (B,D) Details of the grain boundaries and (C,E) corresponding FFT images. (F) Simulated structure of how the epitaxial interface can be constructed between the *sql* 1_Q_-8_L_- and *hcb* 8_L_-10_T_ COF.

The consistent grain boundary angles close to 30° suggest that the relative orientation is not a chance occurrence, but that there is a well-defined molecular interface between the two crystallites that enforces the relative orientation.^17^ This suggests that a high-angle, low-energy boundary is formed, which is also termed the coincidence site lattice. This is not surprising, as a linker system that has a value of α close to α_0_, also has a low lattice mismatch (ε), which is seen as a prerequisite for heteroepitaxy to occur, which is defined as

3 $$\varepsilon =\frac{a_{f}-a_{s}}{a_{f}}$$

where ε is the lattice mismatch and *a*_f_ is the lattice constant of the structure/film grown on the substrate with lattice constant *a*_s_.

While low ε is necessary but not a sufficient criterion for an ideal α value, two COFs with low lattice mismatch can strongly deviate from α_0_.

The lattice mismatch of the 1_Q_-8_L_-10_T_-system calculated from the lattice parameters obtained from PXRD and Pawley refinement is ε = 3.2%. This lattice mismatch is much smaller than the typically required ε < 8% for heteroepitaxy to occur^25^ but much larger than the predicted value, which would be ∼0%.

The relatively large lattice mismatch might also explain why the heteroepitaxy is confined to small regions of the sample, since the buildup of strain in the lattice leads only to island formation or a combined 2D and 3D growth called Stranski–Krastanov mode, which is often seen above 2% mismatch.^26^ Using this model for the epitaxial growth would explain why a small layer of the 1_Q_-8_L_-COF is observed on the surface of the 8_L_-10_T_-COF, since the strain buildup would not allow for a larger growth of the crystallite. This is known from epitaxy as the critical thickness of the layer.

### Nitrogen Adsorption

Finally, nitrogen sorption of the ternary 1_Q_-8_L_-10_T_-COF series showed surprisingly good porosity throughout in all compositions despite the relatively poor crystallinity of some compositions. The pure *hcb* COF has a type 1 isotherm, while the *sql* COF has a clear step in the nitrogen adsorption isotherm at 0.14 *P*/*P*_0_. Starting from the *hcb*-COF, corresponding to 0% 1_Q_, the shape of the isotherm hardly changes, while the total uptake decreases until a composition of 33% 1_Q_ is reached. Starting from 50% 1_Q_, the step at ∼0.14 *P*/*P*_0_ (Figure 8A) becomes clearly visible and more pronounced with higher fractions of 1_Q_. Interestingly, the compositions 50% and 67% 1_Q_ show a higher BET surface area than the pure phase *sql* and *hcb* COFs (Figure 8B).

**Figure 8:**
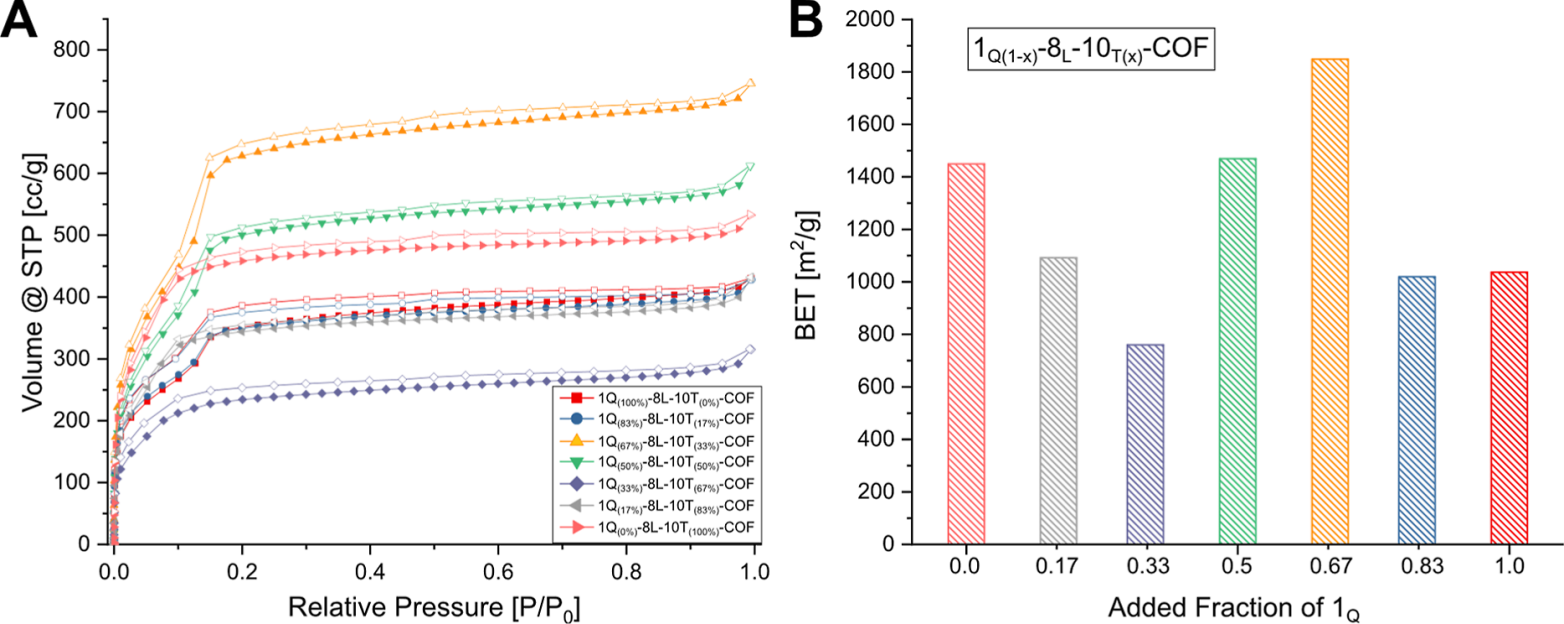
Nitrogen sorption isotherms for the ternary COFs (A). The solid and open symbols represent adsorption and desorption, respectively. Column chart depicts the BET throughout the ternary COF mixtures (B). The color of the column corresponds to the color of the mixtures of the sorption isotherms.

## Conclusions

Our study shows that the linker matching approach can be used to influence the local structure in a ternary COF system. We developed a mathematical model that estimates possible mixing behavior based on linker size compatibility. The finding in TEM images of solid-solutions, heteroepitaxy, and local structures of idealized mixed phases validated the model. No larger, new crystalline phases were detectable, and the reflexes overlap in the PXRD of *hcb* and *sql* COFs. This makes it difficult to systematically optimize the synthesis conditions, as performing TEM measurements for every sample would be too time-consuming. Achieving stoichiometric mixed phases remains challenging without further methods to quantify the individual phases to reach pure mixed phases. Potential strategies to further enhance the formation of ternary COFs either as ordered mixed phases or as solid solutions would match the relative imine bond formation rate of both aldehyde linkers. This could be achieved through the use of modulators, selective protection of one of the aldehydes, or by using electronically different aldehyde precursors. Additionally, preassembling the 4-c, 2-c, and 3-c linkers into a [4 + 2 + 3] trimer could serve as a nucleation site to facilitate more controlled COF growth.

For the 1_Q_-8_L_-10_T_-COF system, the α_exp_ value deviates from the predicted α, and it is not yet clear why this happens. We need to obtain a deeper understanding of this phenomenon, so we could improve the model’s predictions and help identify COF systems with better matching α values, which might allow better mixing and heteroepitaxial growth.

Overall, this work might advance COFs toward understanding and designing mixed-phase systems with tailored properties. This advancement could be particularly interesting for applications requiring precise multiphase structural integration or molecularly defined COF heterojunctions.

## Supplementary Material



## Data Availability

Further TEM data can be accessed at 10.5281/zenodo.14534772.

## Notes

Derivation and calculated values of α; materials, methods, and synthesis details; and additional TEM and XRD data (PDF)
