# Supplementary material for: Evidence of Heteroepitaxy and Solid Solutions in Lattice Matched Ternary Covalent Organic Framework Systems
Source: J Am Chem Soc. 2025 Jun 2;147(23):19702–10. doi: 10.1021/jacs.5c02502 (PMC12164341; doi:10.1021/jacs.5c02502)
Supplement: Supplementary file 1 [file ja5c02502_si_001.pdf]

Supplementary Information
for

Evidence of Heteroepitaxy and Solid Solutions in lattice matched ternary COF Systems

Alena Winter, Juliane Lange, Farzad Hamdi, Panagiotis L. Kastritis, Frederik Haase*

Calculations

Derivation of α_0

We name the corners A, B, C, D, E as shown in Figure S 1. The theoretical position of the chemical molecules in the pentagon predetermines the angles and side lengths.

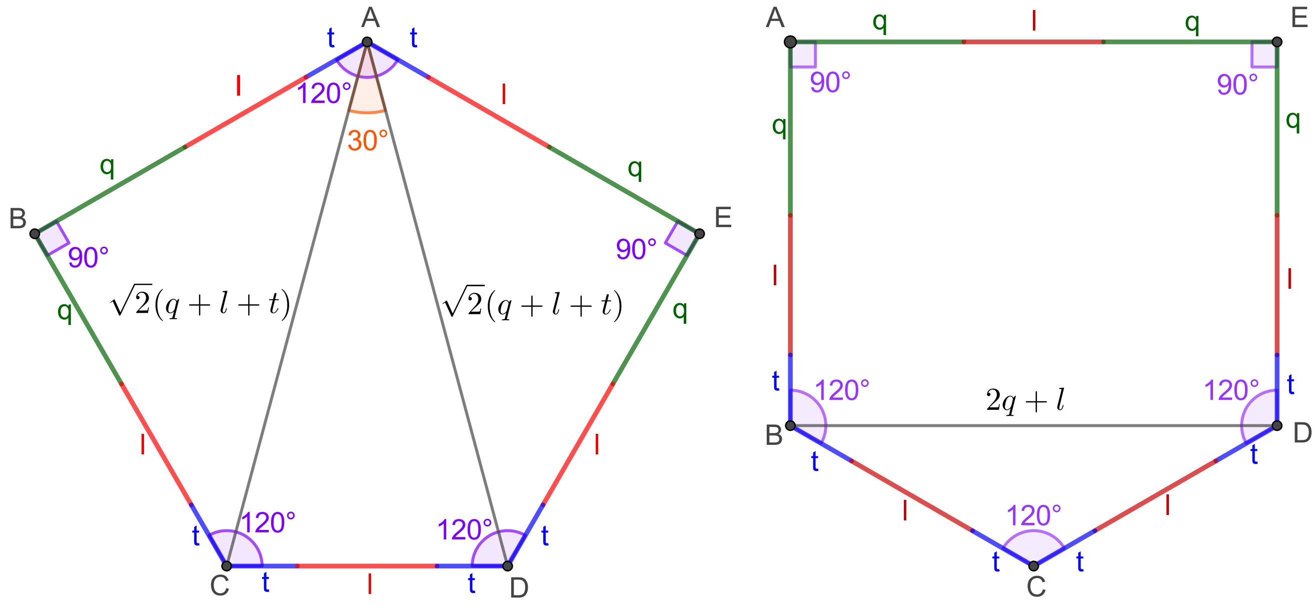

Figure S 1: Sketch of the "Kite"-pentagon (left) and the "Shield"-pentagon (right).

For the "Kite"-pentagon applies:

(1) The following statements pertain to the two isosceles right-angled triangles ΔABC and ΔADE:
- For the two base angles that are not right-angled, the theorem for the sum of angles in a triangle state that they are 45°. In particular, $\sphericalangle CAB = \sphericalangle EAD = 45°$.
- With the Pythagorean theorem follows:

$$|AC|^2 = (q+l+t)^2 + (q+l+t)^2$$
$$|AC|^2 = 2(q+l+t)^2$$
$$|AC| = \sqrt{2}(q+l+t)$$

Equally $|AD| = \sqrt{2}(q+l+t)$.

(2) The result of (1) shows that the triangle ΔACD is isosceles.
- Therefore, the angle $\sphericalangle DAC$ can be calculated from the results of (1).

$$\sphericalangle EAB = \sphericalangle CAB + \sphericalangle DAC + \sphericalangle EAD$$
$$120° = 45° + \sphericalangle DAC + 45°$$
$$\sphericalangle DAC = 30°$$

- By using the law of cosines in the triangle ΔACD, you get:

$$(2t+l)^2 = 2(q+l+t)^2 + 2(q+l+t)^2 - 2\cdot\sqrt{2}(q+l+t)\cdot\sqrt{2}(q+l+t)\cdot\cos(\sphericalangle DAC)$$
$$(2t+l)^2 = 4(q+l+t)^2 - 4(q+l+t)^2\cdot\underbrace{\cos(30°)}_{=\frac{\sqrt{3}}{2}}$$
$$(2t+l)^2 = \left(4-2\sqrt{3}\right)(q+l+t)^2$$
$$\frac{(2t+l)^2}{(q+l+t)^2} = 4-2\sqrt{3}$$

The expected value for α_0 is obtained by taking the square root. The negative solution can be ignored as the lengths are $q, l, t \geq 0$.

For the "Shield"-pentagon applies:

- By using the law of cosines in the isosceles triangle ΔBCD, you get

$$(2q + l)^2 = (2t + l)^2 + (2t + l)^2 - 2(2t + l)(2t + l) \cdot \cos(\sphericalangle BCD)$$

$$(2q + l)^2 = 2(2t + l)^2 - 2(2t + l)^2 \cdot \underbrace{\cos(120°)}_{=-\frac{1}{2}}$$

$$(2q + l)^2 = 3(2t + l)^2$$

When taking the square root, the negative solution can be ignored as the lengths are $q, l, t \geq 0$.

- The equivalent ratio is obtained by rearranging the equation.

$$2q + l = \sqrt{3}(2t + l)$$

$$2q + l = 2\sqrt{3}t + \sqrt{3}l \qquad | - \sqrt{3}l$$

$$2q + (1 - \sqrt{3})l = 2\sqrt{3}t \qquad |: 2\sqrt{3}$$

$$\frac{1}{\sqrt{3}}q + \frac{\sqrt{3} - 3}{6}l = t \qquad | \cdot \left(2 - \sqrt{4 - 2\sqrt{3}}\right)$$

$$\sqrt{4 - 2\sqrt{3}}q + \left(\sqrt{4 - 2\sqrt{3}} - 1\right)l = 2t - \sqrt{4 - 2\sqrt{3}}t$$

$$\sqrt{4 - 2\sqrt{3}}(q + l + t) = 2t + l$$

$$\sqrt{4 - 2\sqrt{3}} = \frac{2t + l}{q + \mathrm{l} + t}$$

This means that the ideal ratio is the same for both pentagons and we call it α_0.

$$\alpha_0 := \frac{2t + l}{q + \mathrm{l} + t} = \sqrt{4 - 2\sqrt{3}} \approx 0.732$$

The term $\sqrt{4 - 2\sqrt{3}}$ can be simplified to $\sqrt{3} - 1$ by using the binomial formula:

$$\sqrt{4 - 2\sqrt{3}} = \sqrt{3 - 2\sqrt{3} + 1} = \sqrt{\sqrt{3}^2 - 2\sqrt{3} + 1} = \sqrt{\left(\sqrt{3} - 1\right)^2} = \sqrt{3} - 1$$

Derivation of α_{exp}

We define the length of the unit cell by a with the respective indices for square (*sql*) and hexagonal (*hcb*) lattice.

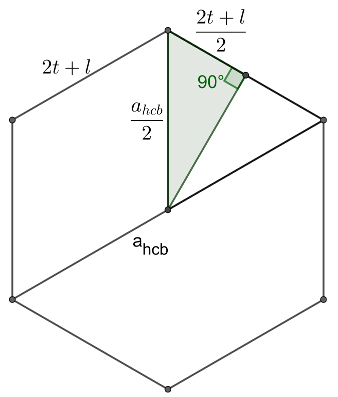

Figure S 2: Sketch of the right-angled auxiliary triangle in the hexagonal lattice.

If we write the measurable size of the unit cell with the lengths q, l, t of the linkers, we obtain two equations.

$$I \; 2q + l = a_{sql}$$

$$II \; 2t + l = \frac{a_{hcb}}{\sqrt{3}}$$

Equation II follows after setting up a right-angled auxiliary triangle (Figure S 2) and using the tangent.

$$\underbrace{\tan(30°)}_{=\frac{1}{\sqrt{3}}} = \frac{\frac{2t+l}{2}}{\frac{a_{hcb}}{2}} = \frac{2t+l}{a_{hcb}}$$

If you add the two equations, you get:

$$I + II \; 2q + 2l + 2t = a_{sql} + \frac{a_{hcb}}{\sqrt{3}} \qquad |:2$$

$$q + l + t = \frac{\sqrt{3}a_{sql} + a_{hcb}}{2\sqrt{3}}$$

If the two equations II and $I + II$ are inserted into the ratio α, an equation is obtained that allows to calculate α from the sizes of the unit cells of the two pure COFs.

$$\alpha = \frac{2t+l}{q+\mathrm{l}+t} = \frac{2\sqrt{3}\frac{a_{hcb}}{\sqrt{3}}}{\sqrt{3}a_{sql} + a_{hcb}} = \frac{2\,\mathrm{a}_{hcb}}{\sqrt{3}\,\mathrm{a}_{sql} + \mathrm{a}_{hcb}}$$

This experimental alpha is called α_{exp}.

$$\alpha_{\mathrm{exp}} := \frac{2\,\mathrm{a}_{hcb}}{\sqrt{3}\,\mathrm{a}_{sql} + \mathrm{a}_{hcb}}$$

Calculation of α_0 for all linker combinations

Based on the formula above the lengths of the individual building blocks and building block fragments was determined from the sizes of force field optimized molecular fragments and based on simple geometric considerations. All building blocks were forced to be two dimensional. For the calculations of α only the absolute length of the building block was considered and angles of attachment and offsets of the linker were ignored (Figure S 3, Table S 1). The position of the nitrogen within the imine is

arbitrary for the calculation. The systems that match the desired α_0 values the closest are listed in Table S 2 below.

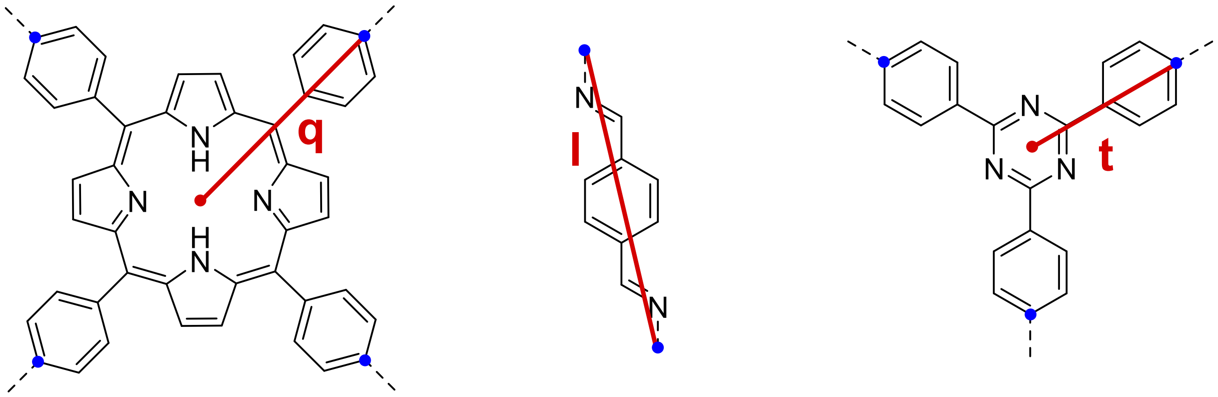

Figure S 3: Graphical indication of how q, l and t are defined.

Table S 1: Lengths of building blocks. The blue dots indicate the points of attachment.

Building block	Desig-nation	Length (Å)
	**q**	
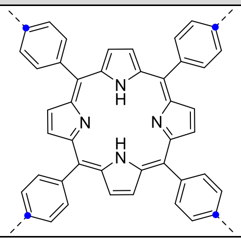	1	7.856
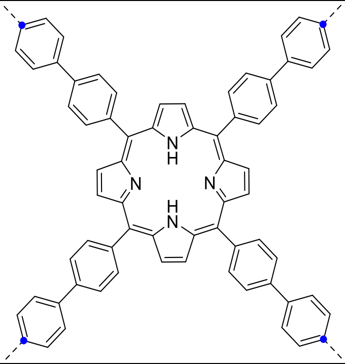	2	12.24
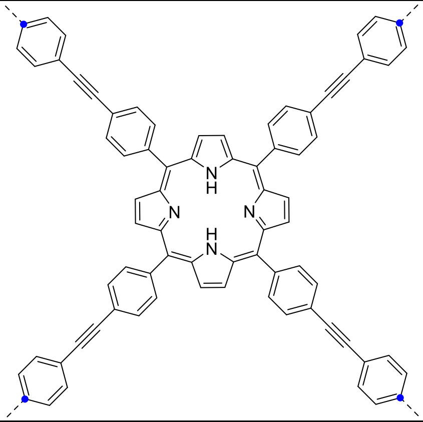	3	14.98
	**l**	
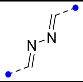	4	6.918
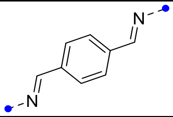	5	10.35
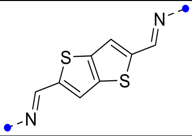	6	11.9
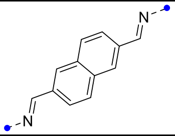	7	12.72

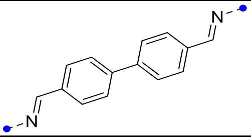	8	14.68
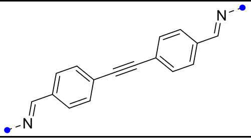	9	17.4
	**t**	
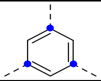	10	1.425
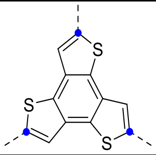	11	2.846
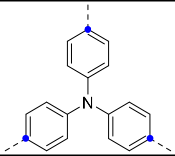	12	4.387
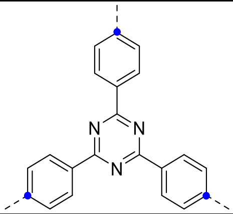	13	5.812

Table S 2: List of calculates α values in descending order based on the deviation from α defined as $|\alpha_0 - \alpha|$.

**q (1-3)**	**l (4-9)**	**t (10-13)**		**α**	$|\alpha_0 - \alpha|$
			**α_0:**	**0.732051**	
**2**	7	12		0.73229	0.000239652
**1**	8	10		0.73164	0.00041041
**3**	7	13		0.726387	0.005663338
**2**	6	12		0.724658	0.007392578
**3**	8	13		0.741565	0.009514502
**2**	4	13		0.742485	0.010434684
**3**	6	13		0.71958	0.012470834
**1**	4	11		0.71567	0.016381139
**2**	8	12		0.749115	0.017063889
**3**	9	12		0.711876	0.020174967
**2**	9	11		0.710749	0.021301409
**2**	5	12		0.708873	0.023177726
**1**	7	10		0.707632	0.024418993
**3**	5	13		0.705652	0.026398917
**1**	9	10		0.758953	0.026901793
**3**	9	13		0.75994	0.027888863
**1**	5	11		0.762072	0.030021237
**1**	6	10		0.696405	0.035645986
**2**	9	12		0.769134	0.03708321
**2**	5	13		0.773634	0.041583597
**3**	8	12		0.6889	0.043151091
**1**	6	11		0.778356	0.046305175
**2**	8	11		0.684369	0.047681741
**2**	6	13		0.785324	0.053273488
**1**	7	11		0.786055	0.054004229
**2**	7	13		0.790999	0.05894853
**1**	5	10		0.672486	0.059564934
**3**	7	12		0.669817	0.062233515
**3**	4	13		0.669149	0.062901913
**2**	4	12		0.666389	0.06566229
**2**	7	11		0.66203	0.070020876
**1**	8	11		0.80264	0.07058943
**3**	6	12		0.661228	0.070822858
**2**	8	13		0.803562	0.071511484
**3**	9	11		0.655523	0.076527931
**2**	6	11		0.651845	0.080206303
**2**	9	10		0.651778	0.08027268
**2**	9	13		0.818607	0.086556153
**1**	4	12		0.818958	0.08690767
**3**	5	12		0.643595	0.088455939
**1**	9	11		0.821711	0.089659916

**2**	5	11		0.630676	0.101374656
**3**	8	11		0.626751	0.105299988

Experimental

Materials

All linker molecules were obtained from BLD Pharm, abcr, and TCI. Solvents and Acetic acid were obtained from Fischer Scientific and Sigma Aldrich.

COF synthesis

The percentage refers to the ratio between the aldehydes.

Synthesis of $1_{Q(100-X\%)}$-8_L-$1_{T(X\%)}$-COFs

General Procedure: Aldehydes were weighed into a 50 mL crimp vial according to Table 1. *o*-Dichlorobenzene (*o*-DCB) was added and the vial was stirred at 90 °C until the aldehydes were dissolved. The vial was removed from the heat and Benzidine (BDA), *n*-butanol (*n*-BuOH) and 3 M acetic acid (AcOH) were quickly added to the vial. The vial was closed and placed in the ultrasonic bath for 10 min. The mixture was heated in an oven at 120 °C for 3 days. After being cooled to room temperature, the solvent was filtered of and the solid was washed thoroughly with methanol (MeOH). Soxhlet extraction with MeOH overnight followed by supercritical CO_2 drying yielded the corresponding COFs.

Synthesis of $1_{Q(100-X\%)}$-5_L-$1_{T(X\%)}$-COFs

General Procedure: Aldehydes were weighed into a 50 mL crimp vial according to Table 2. *o*-Dichlorobenzene (*o*-DCB) was added and the vial was stirred at 90 °C until the aldehydes were dissolved. The vial was removed from the heat and Phenylenediamine (PDA), *n*-butanol (*n*-BuOH) and 3 M acetic acid (AcOH) were quickly added to the vial. The vial was closed and placed in the ultrasonic bath for 10 min. The mixture was heated in an oven at 120 °C for 3 days. After being cooled to room temperature, the solvent was filtered of and the solid was washed thoroughly with methanol (MeOH). Soxhlet extraction with MeOH overnight followed by supercritical CO_2 drying yielded the corresponding COFs. 1_Q-5_L-10_T - System

Crystallinity of the pure phase samples were confirmed by PXRD (Figure 4). Pawley refinement yielded for the 10_T-5_L-COF with a *hcb* lattice and *P6/m* symmetry refined parameters of *a* = *b* = 21.7072 Å, *c* = 3.45 Å, with angles α = β = 90° and γ = 120°. For the 1_Q-5_L-COF with a *sql* lattice and a *P4* symmetry, the refinement resulted in *a* = *b* = 24.9721 Å, *c* = 4.77 Å, with α = β = γ = 90°. The α_{exp} based on the 1_Q-5_L- and 5_L-10_T -COFs is 0.6683, which is close to the predicted value of 0.6725. This shows that this system deviates significantly from the ideal α_0.

Table S 3: List of PDA-COF based synthesis

Name	1 (%)	10 (%)	2θ (°)	FWHM (Å)	d-spacing (Å)
$1_{100\%}$-5-$10_{0\%}$-COF	100	0	3.506396	0.47244552	25.19750349
$1_{83\%}$-5-$10_{17\%}$-COF	83	17	3.480978	0.4112516	25.38143787
$1_{67\%}$-5-$10_{33\%}$-COF	67	33	3.563012	0.4468113	24.79724337
$1_{50\%}$-5-$10_{50\%}$-COF	50	50	3.464063	0.52546336	25.50533667
$1_{33\%}$-5-$10_{67\%}$-COF	33	67	4.11722	1.05660242	21.46051103
$1_{17\%}$-5-$10_{83\%}$-COF	17	83	4.470362	0.79072998	19.76597112
$1_{0\%}$-5-$10_{100\%}$-COF	0	100	4.669263	0.51131205	18.92441568

Table S 5: List of BDA-COF based synthesis conditions.

Sample	PTT		TFB		BDA		Solvent		Acid	Yield
	m [mg]	n [µmol]	m [mg]	n [µmol]	m [mg]	n [mmol]	V(*o*-DCB) [mL]	V(*n*-BuOH) [mL]	V(3 M AcOH) [mL]	[%]
PTT_{100}-BDA-TFB_{0}-COF	54.51	75.0	–	–	27.64	0.15	2.5	2.5	0.5	79.1
$PTT_{83.33}$-BDA-$TFB_{16.67}$-COF	45.42	62.5	2.70	16.7	27.64	0.15	2.5	2.5	0.5	65.9
$PTT_{66.67}$-BDA-$TFB_{33.33}$-COF	43.61	60.0	6.48	40.0	33.16	0.18	3.0	3.0	0.6	65.2
PTT_{50}-BDA-TFB_{50}-COF	32.71	45.0	9.73	60.0	33.16	0.18	3.0	3.0	0.6	68.5
$PTT_{33.33}$-BDA-$TFB_{66.67}$-COF	25.44	35.0	15.13	93.3	38.69	0.21	3.5	3.5	0.7	53.9
$PTT_{16.67}$-BDA-$TFB_{83.33}$-COF	14.54	20.0	21.62	133	44.22	0.24	4.0	4.0	0.8	63.5
PTT_{0}-BDA-TFB_{100}-COF	–	–	29.19	180	49.47	0.27	4.5	4.5	0.9	56.2

Table S 4: List of PDA-COF based synthesis conditions

Sample	PTT		TFB		PDA		Solvent		Acid	Yield
	m [mg]	n [µmol]	m [mg]	n [µmol]	m [mg]	n [mmol]	V(*o*-DCB) [mL]	V(*n*-BuOH) [mL]	V(3 M AcOH) [mL]	[%]
PTT_{100}-PDA-TFB_{0}-COF	64.41	90.0	–	–	19.45	0.18	3.0	3.0	0.6	95.0
$PTT_{83.33}$-PDA-$TFB_{16.67}$-COF	54.51	75.0	3.24	20.0	19.45	0.18	3.0	3.0	0.6	82.9
$PTT_{66.67}$-PDA-$TFB_{33.33}$-COF	50.88	70.0	6.48	40.0	22.69	0.21	3.5	3.5	0.7	77.8
PTT_{50}-PDA-TFB_{50}-COF	43.61	60.0	12.97	80.0	25.94	0.24	4.0	4.0	0.8	70.9
$PTT_{33.33}$-PDA-$TFB_{66.67}$-COF	32.70	45.0	19.46	120	29.18	0.27	4.5	4.5	0.9	73.1
$PTT_{16.67}$-PDA-$TFB_{83.33}$-COF	18.17	25	27.02	167	32.42	0.30	5.0	5.0	1.0	73.6
PTT_{0}-PDA-TFB_{100}-COF	–	–	22.70	140	22.70	0.21	3.5	3.5	0.7	Quant.

PXRD data

	2θ	*hkl* reflections
10_T-8_L-COF	3.62°, 6.23°, 7.18°	100, 2-10, 200
1_Q-8_L-COF	3.08°, 5.98°	100, 010
10_T-5_L-COF	4.84°, 8.41°, 9.65°	100, 110, 200
1_Q-5_L-COF	3.67°, 7.21°	100, 200

Methods

N_2-sorption: All N_2-sorption measurements were carried out using a Quantachrome autosorb iQ2. Before gas adsorption measurements, the sample was activated by drying under a vacuum at 120 °C for 16 h. The resulting sample was then used for gas adsorption measurements from 0 to 1 atm at 77 K. The Brunauer-Emmett-Teller (BET) method was utilized to calculate the specific surface areas. By using the non-local density function theory model, the pore size distribution curves were derived from the sorption data.

PXRD: High-resolution synchrotron X-ray diffraction and total scattering measurements were performed at beamline ID31 at the European Synchrotron Radiation Facility (ESRF). The sample powders were loaded into cylindrical slots (approx. 1 mm thickness) held between Kapton windows in a high-throughput sample holder. Each sample was measured in a transmission with an incident X-ray energy of 75.00 keV (λ = 0.1653 Å). Measured intensities were collected using a Pilatus CdTe 2M detector (1679 × 1475 pixels, 172 × 172 μm^2 each) positioned with the incident beam in the corner of the detector. The sample-to-detector distance was approximately 1.5 m for the high-resolution measurements. Background measurements for the empty windows were measured and subtracted. NIST SRM 660b (LaB_6) was used for geometry calibration performed with the software pyFAI followed by image integration including a flat-field, geometry, solid-angle, and polarization corrections. SAXS measurements were additionally performed using a PerkinElmer detector (2048 × 2048 pixels, 200 × 200 μm^2 each) at a distance of 8.9 m with the beam roughly centered on the detector and a flight tube to minimize air scattering. Low resolution PXRD-measurements were conducted with an Incoatec (Geesthacht, Germany) IµS equipped with a microfocus source and a monochromator for CuKα radiation (λ = 1.54 Å). The 2D scattering patterns were captured using a Vantec 500 2D detector (Bruker AXS, Karlsruhe). Moving the detector allowed acquisition of diffractograms spanning both small and wide angles.

TEM: The COF sample was suspended in *n*-butanol at a concentration of 2 mg/mL. This suspension underwent a 10-minute sonication process at room temperature in a standard ultrasonic bath (Bandelin Sonorex Digiplus - Bandelin Electronic GmbH & Co. KG) and was subsequently centrifuged at 1,000 g for 5 minutes (Heraeus Fresco 21 - ThermoFisher Scientific). Following this, a 3.5 µl aliquot of the supernatant *n*-butanol was dispensed onto the carbon side of lacey grids, which were already laid on 525-type ashless filter paper to remove excess solution. This technique enables the lacey film to selectively filter fine COF particles. Subsequently, the grids were air-dried and then securely affixed within the ThermoFisher Autogrid assembly using the standard tools, all conducted at room temperature.

The prepared samples were loaded into a Thermo Fisher Scientific Glacios cryogenic electron microscope, operating at 200 kV and equipped with a Falcon 4i direct electron detector. These samples were left to equilibrate overnight under vacuum conditions within the microscope's autoloader at room temperature to ensure thorough drying. Following this, the samples were gradually cooled to cryogenic temperatures (below 100°K) inside the microscope while maintaining the vacuum.

For imaging, a low-dose strategy was employed. Images were captured with an average electron dose of 50 e/Å² at a pixel size of 0.936 Å in electron counting mode, and a frame rate of 310/s. The electron event data underwent motion correction and were stored as a single frame using the on-the-fly frame alignment feature of the Falcon 4i camera.

Supplementary Figures

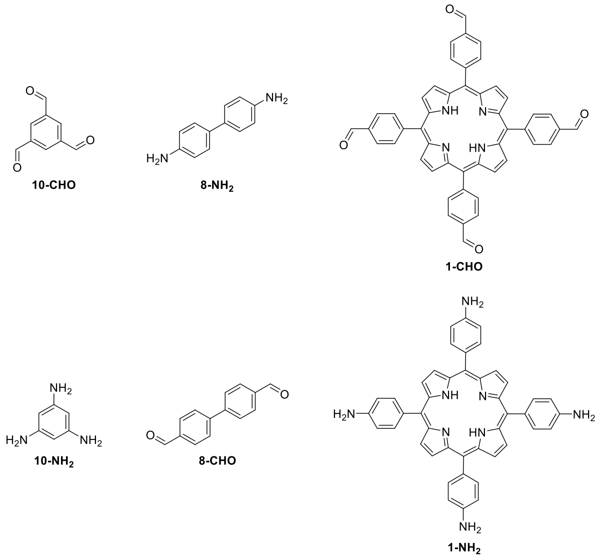

Figure S 4: Schematic depiction of the molecules for the ternary COF system, which would give a contrary orientation of the imine bond.

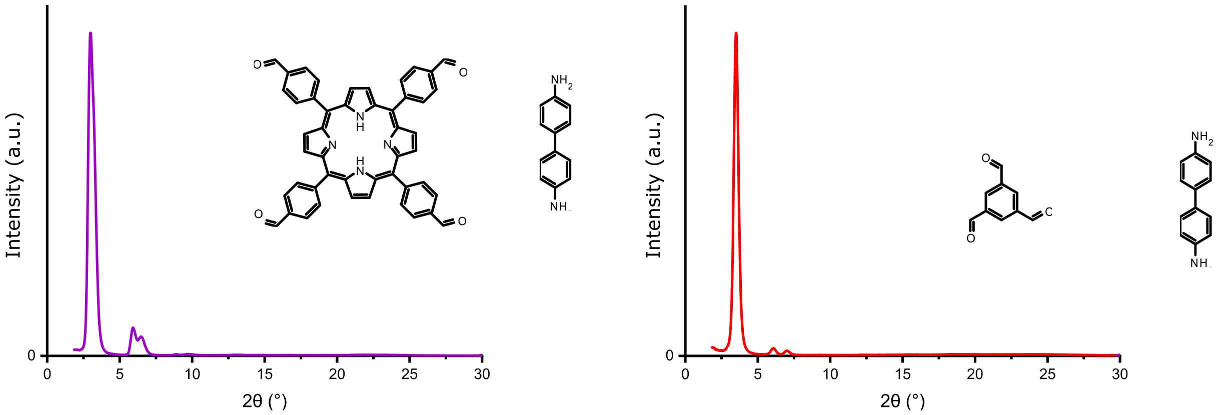

Figure S 5: PXRD Diffractograms (Cu Kα$_1$) for the individually optimized synthesis conditions for 1_Q-8_L-COF (left) and 10_T-8_L-COF (right).

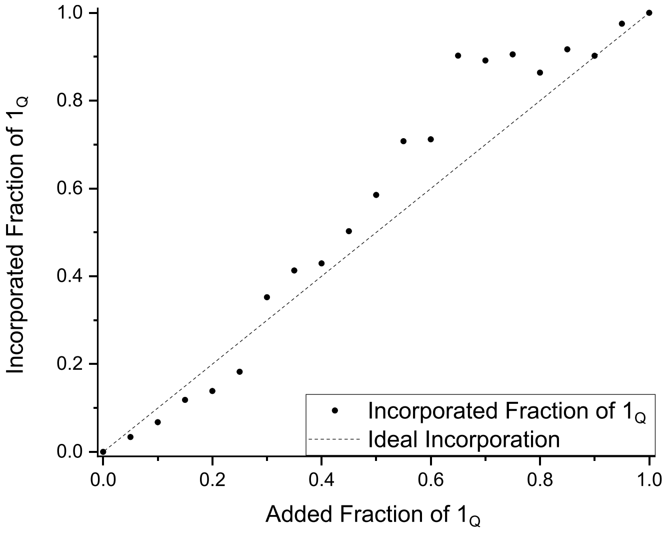

Figure S 5: Incorporated Fraction of 1_Q in ternary mixtures compared to the theoretical added fraction of 1_Q. Incorporated amount was determined by ^{1}H-NMR and the integration of aldehyde peaks of 1_Q and 10_T of digested COF samples.

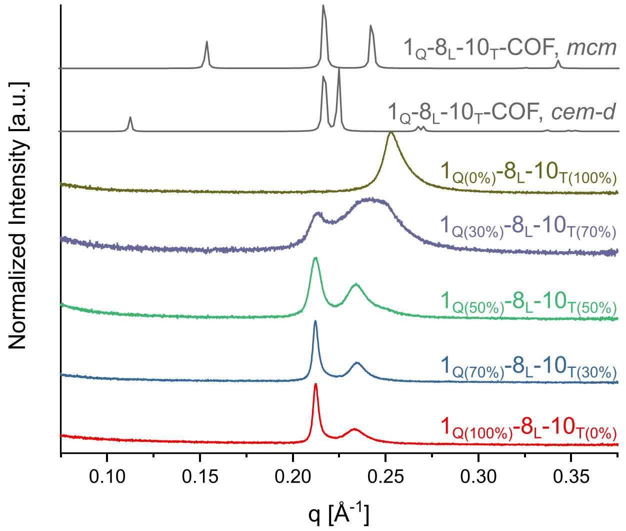

Figure S 6: Synchrotron SAXS (λ = 0.1653 Å) of the ternary COF series and comparison with simulated PXRD for cem-d and mcm.

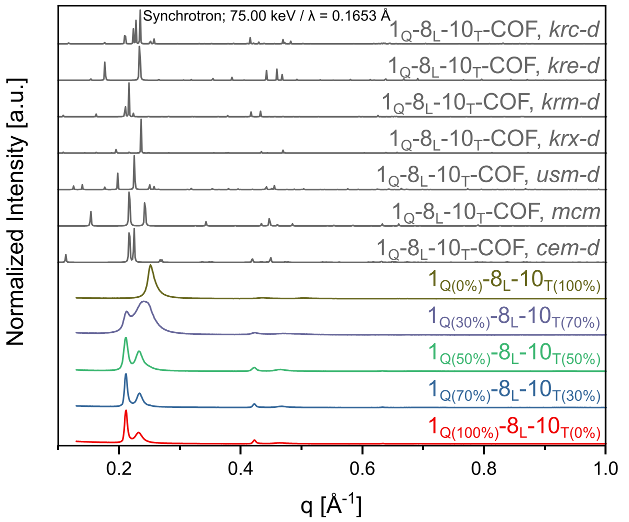

Figure S 8: HR-PXRD data (λ = 0.1653 Å) compared to the simulated PXRD for the in nets shown in Figure 1.

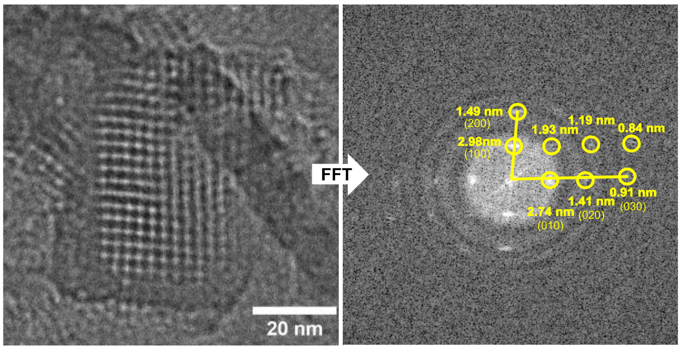

Figure S 7: TEM image of a sql 1_Q-8_L-COF crystallite (left) and the corresponding FFT (right) in the $1_{Q(50\%)}$-8_L-$10_{T(50\%)}$-COF sample.

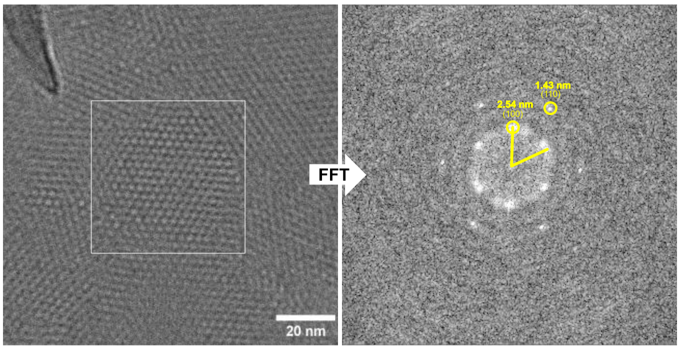

Figure S 8: TEM image of a hcb 10_T-8_L-COF crystallite (left) and the corresponding FFT (right) in the $1_{Q(50\%)}$-8_L-$10_{T(50\%)}$-COF sample.

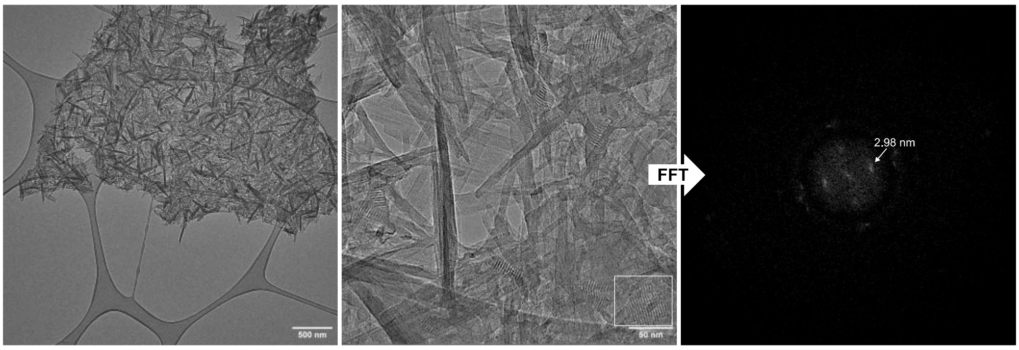

Figure S 9: "Haystack" morphology in 13 500x (left) and 150 000x (middle) magnification, FFT of the highlighted area (right).

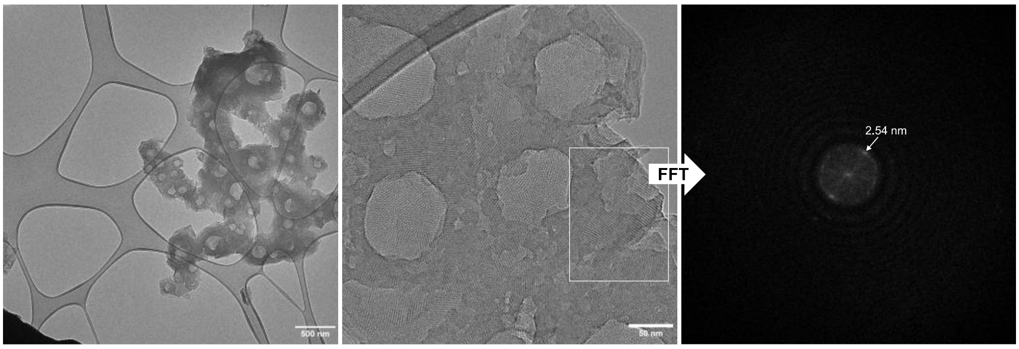

Figure S 10: "Hollow Spheres" in 13 500x (left) and 150 000x (middle) magnification, FFT of the highlighted area (right).

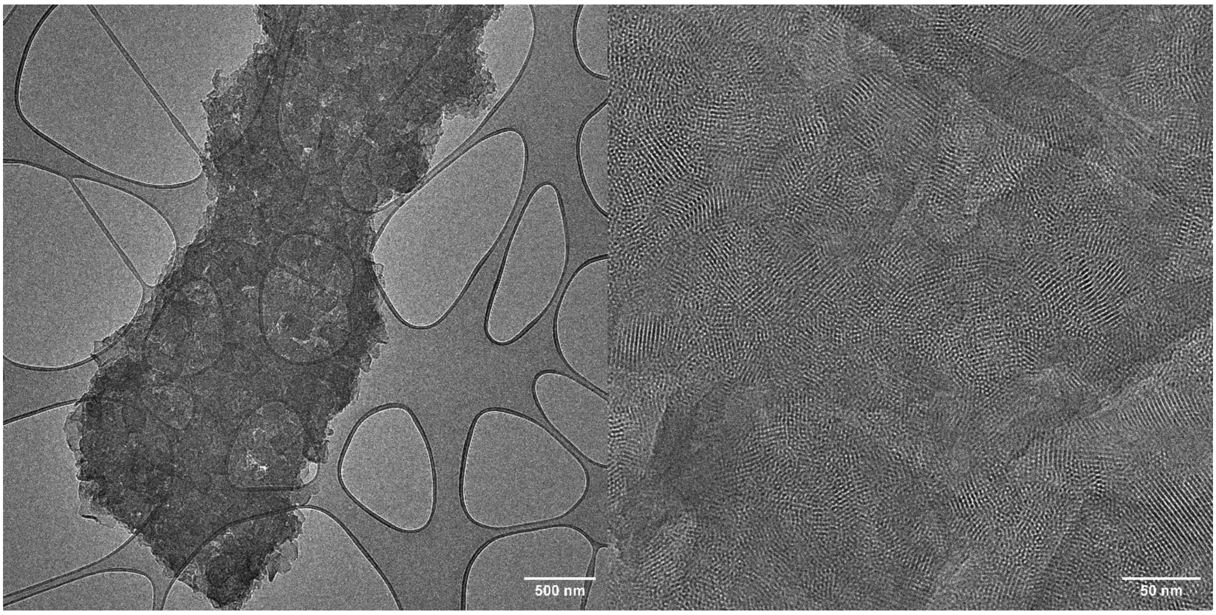

Figure S 11: "Intergrown Crystallites" in 13 500x (left) and 150 000x (right) magnification.

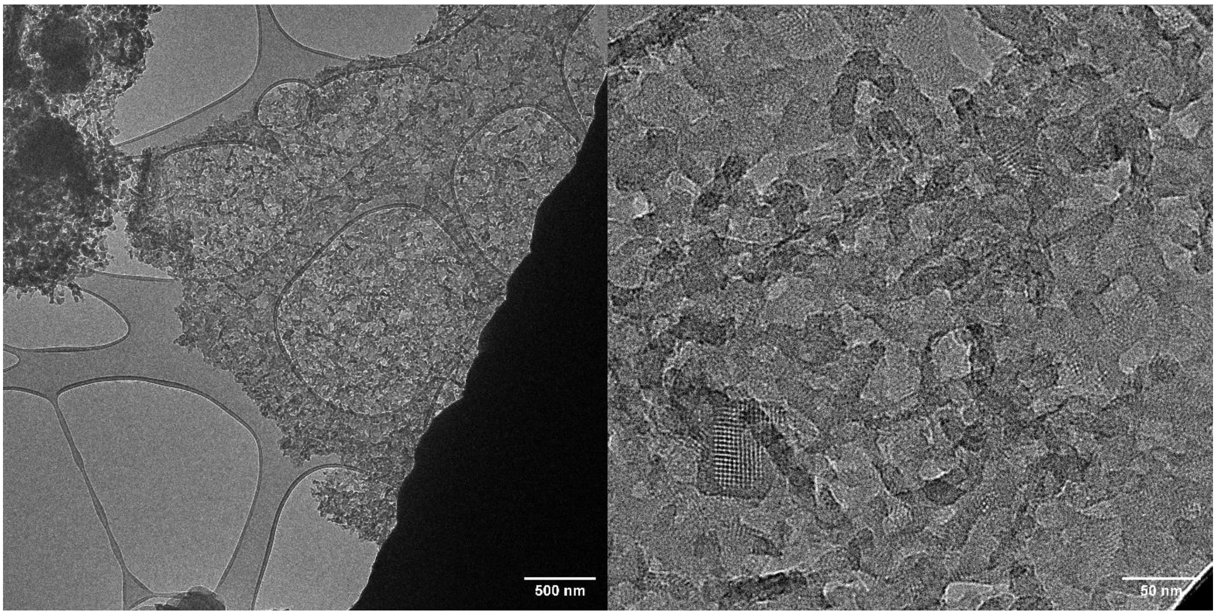

Figure S 12: "Intergrown Crystallites" morphology in 13 500x (left) and 150 000x (right) magnification.

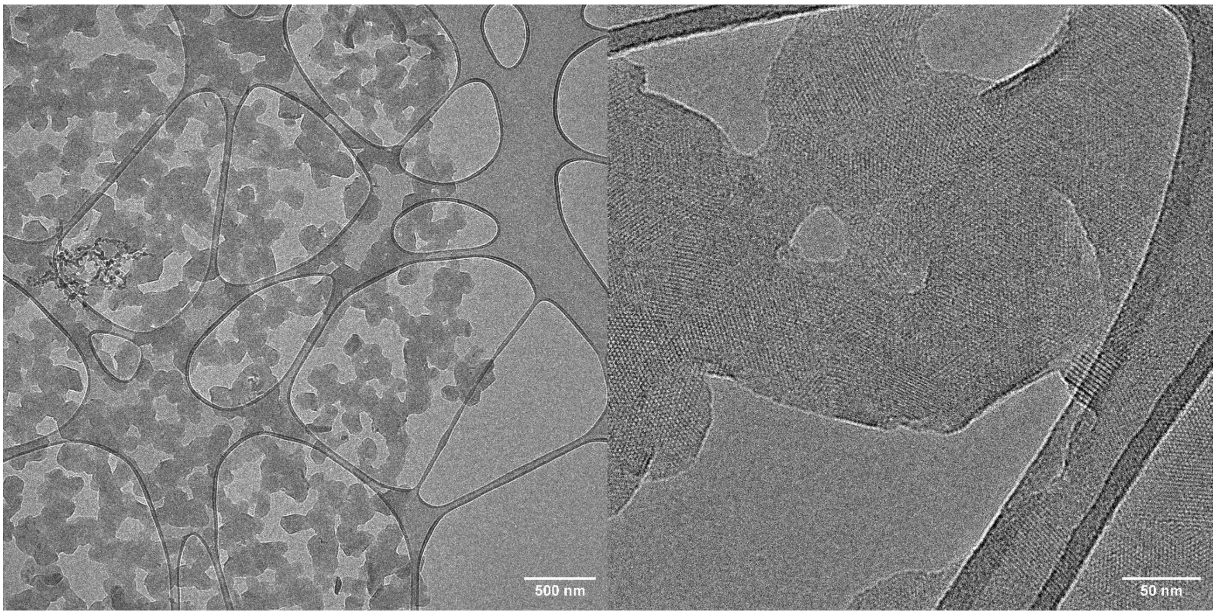

Figure S 13: "Dilute Network" morphology in 13 500x (left) and 150 000x (right) magnification.

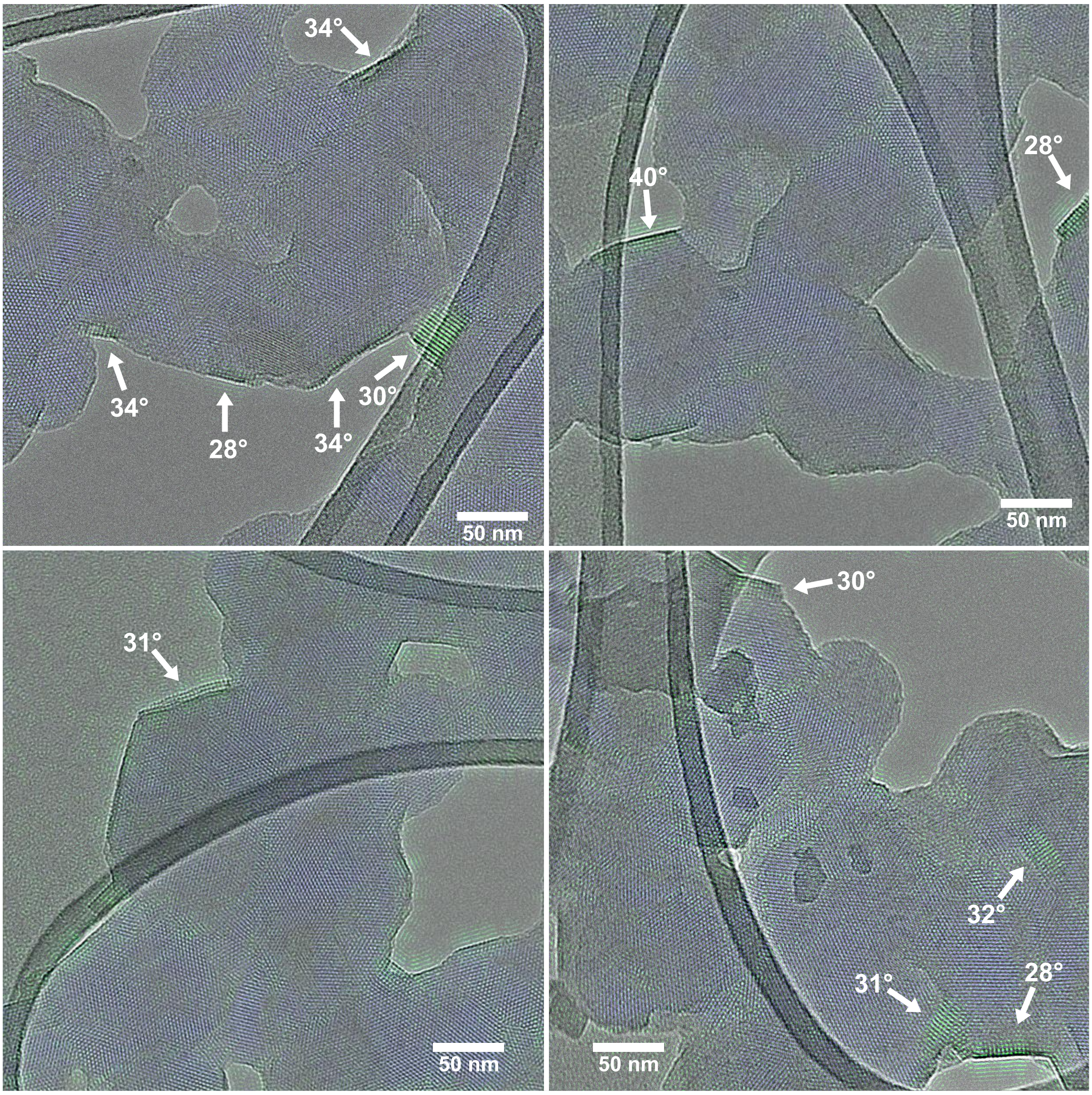

Figure S 14: TEM images of well aligned sql and hcb crystallites with heteroepitaxial grain boundaries within the $1_{Q(50\%)}$-8_L-$10_{T(50\%)}$-COF sample. d-spacings that can be assigned to the sql and the hcb phase are highlighted through a Fourier filtered overlay in green and blue, respectively.

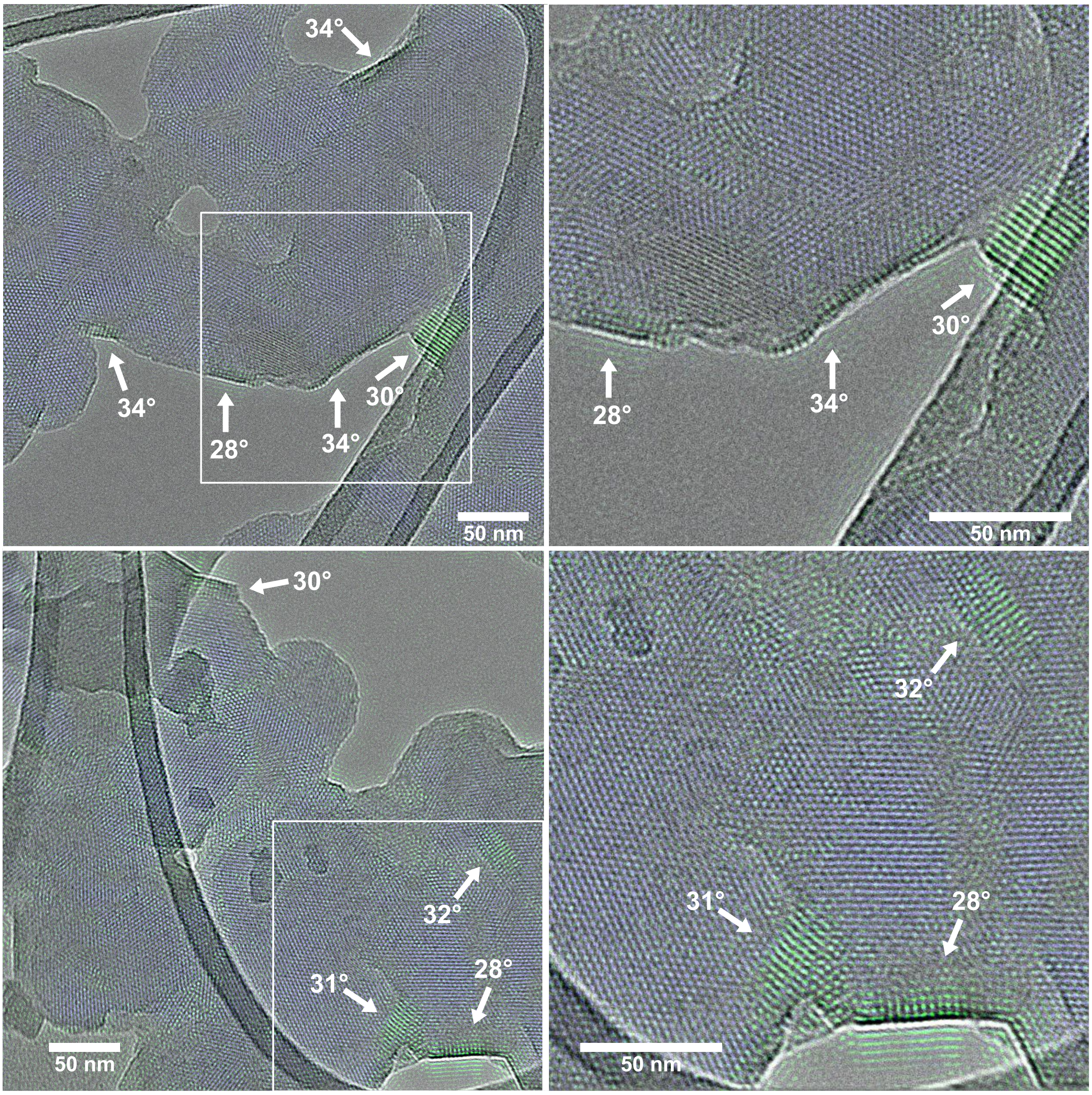

Figure S 15: Details of TEM images from Figure S 14 *of well aligned sql and hcb crystallites with heteroepitaxial grain boundaries within the $1_{Q(50\%)}$-8_L-$10_{T(50\%)}$-COF sample. d-spacings that can be assigned to the sql and the hcb phase are highlighted through a Fourier filtered overlay in green and blue, respectively.*

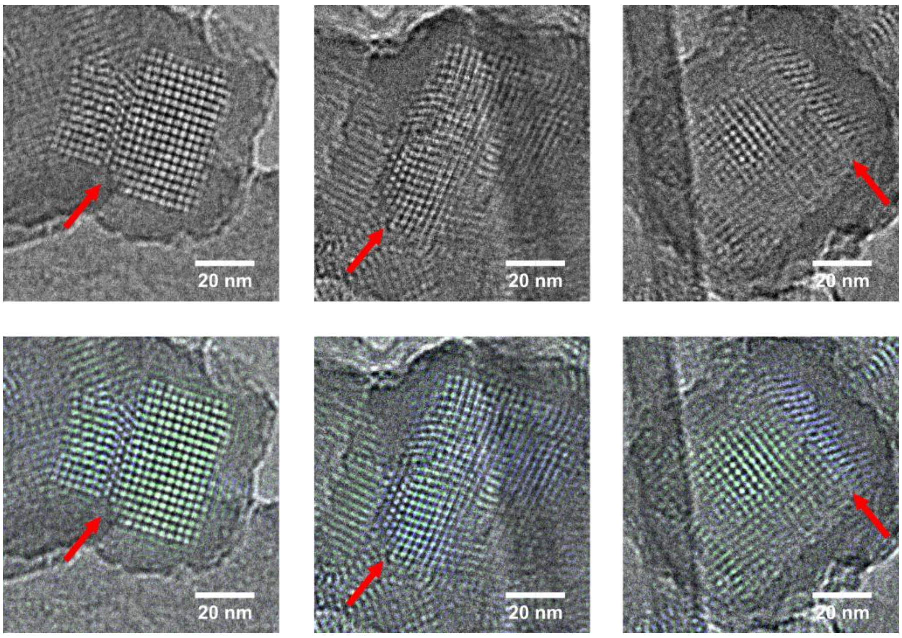

Figure S 18: TEM images of well resolved heteroepitaxial interfaces between hcb and sql phases of within the $1_{Q(50\%)}$-8_{L}-$10_{T(50\%)}$-COF sample.

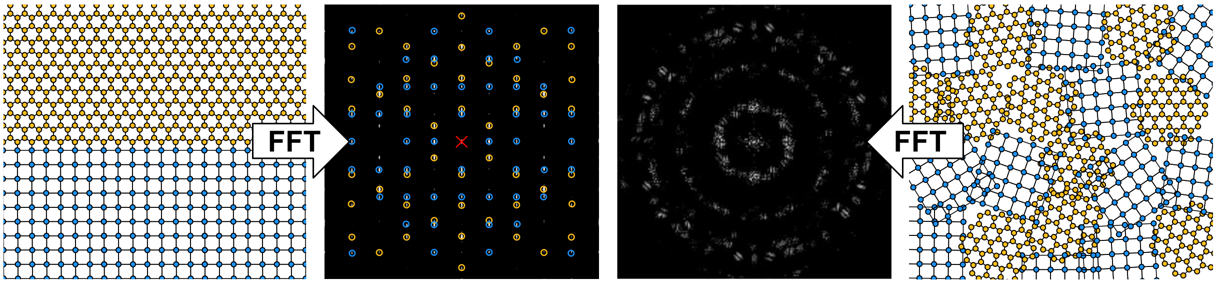

Figure S 16: Schematic images of the expected FFT patterns for an idealized heteroepitaxial interface and randomly oriented crystallites.

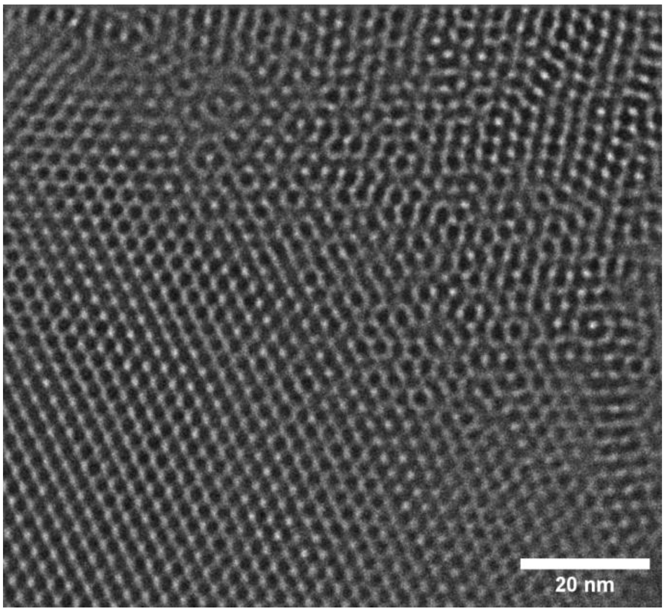

Figure S 17: Example of Moiré generated by two overlapping hexagonal crystallites in the TEM image of $1_{Q(50\%)}$-8_L-$10_{T(50\%)}$-COF sample.

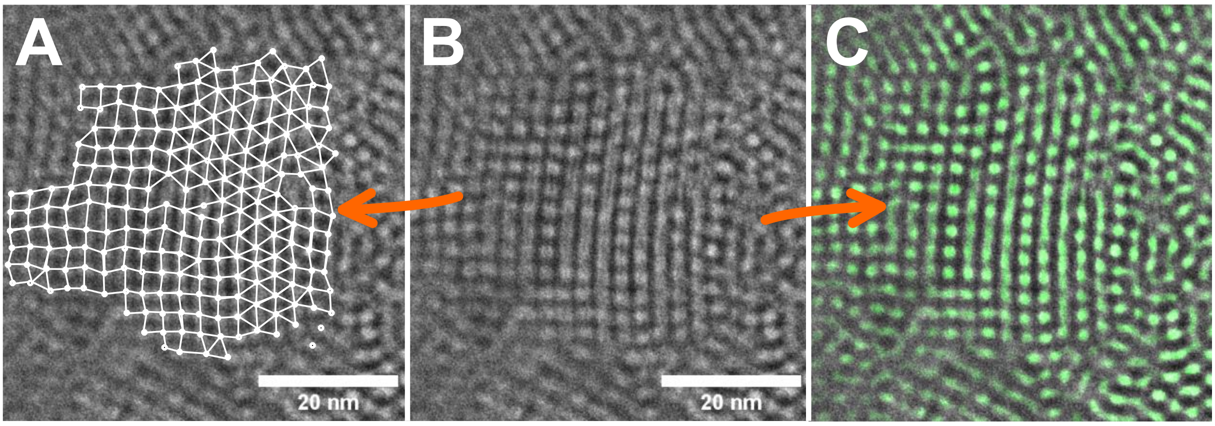

Figure S 18: TEM image of a sql-hcb phase boundary with in one crystallite in the $1_{Q(50\%)}$-8_L-$10_{T(50\%)}$-COF sample.

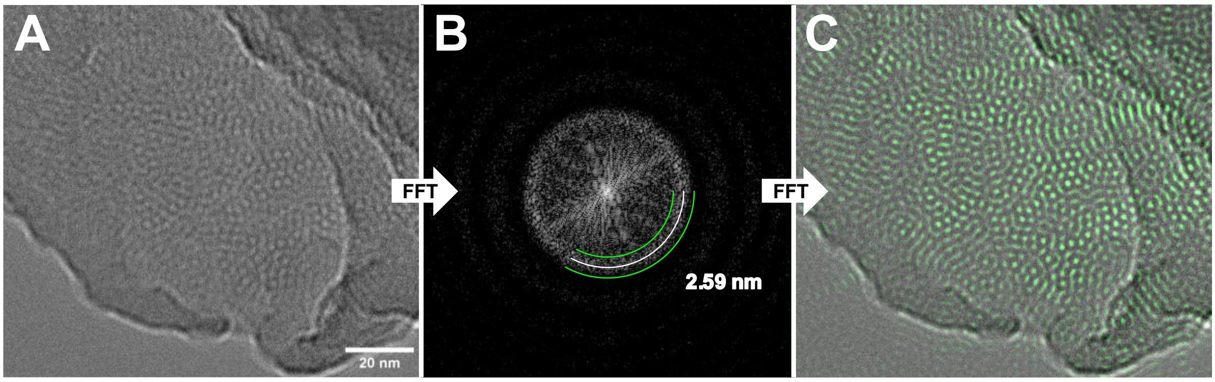

Figure S 19: TEM image of a random pore structure (A) with the corresponding Fourier Transform Image (B). Fourier filtered image of the ring observed in the Fourier transform overlaid over the TEM image. Contrast transfer function (CTF) rings are ruled out based on radial integration, while the complete drying of the sample under high vacuum excludes ice rings.

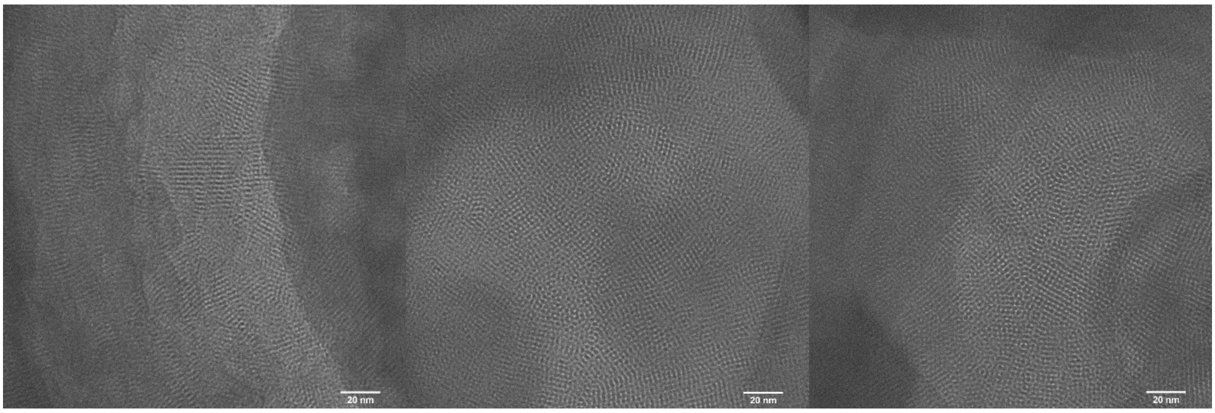

Figure S 20: TEM image of mixed phases of $1_{Q(50\%)}$-5_L-$10_{T(50\%)}$-COF.

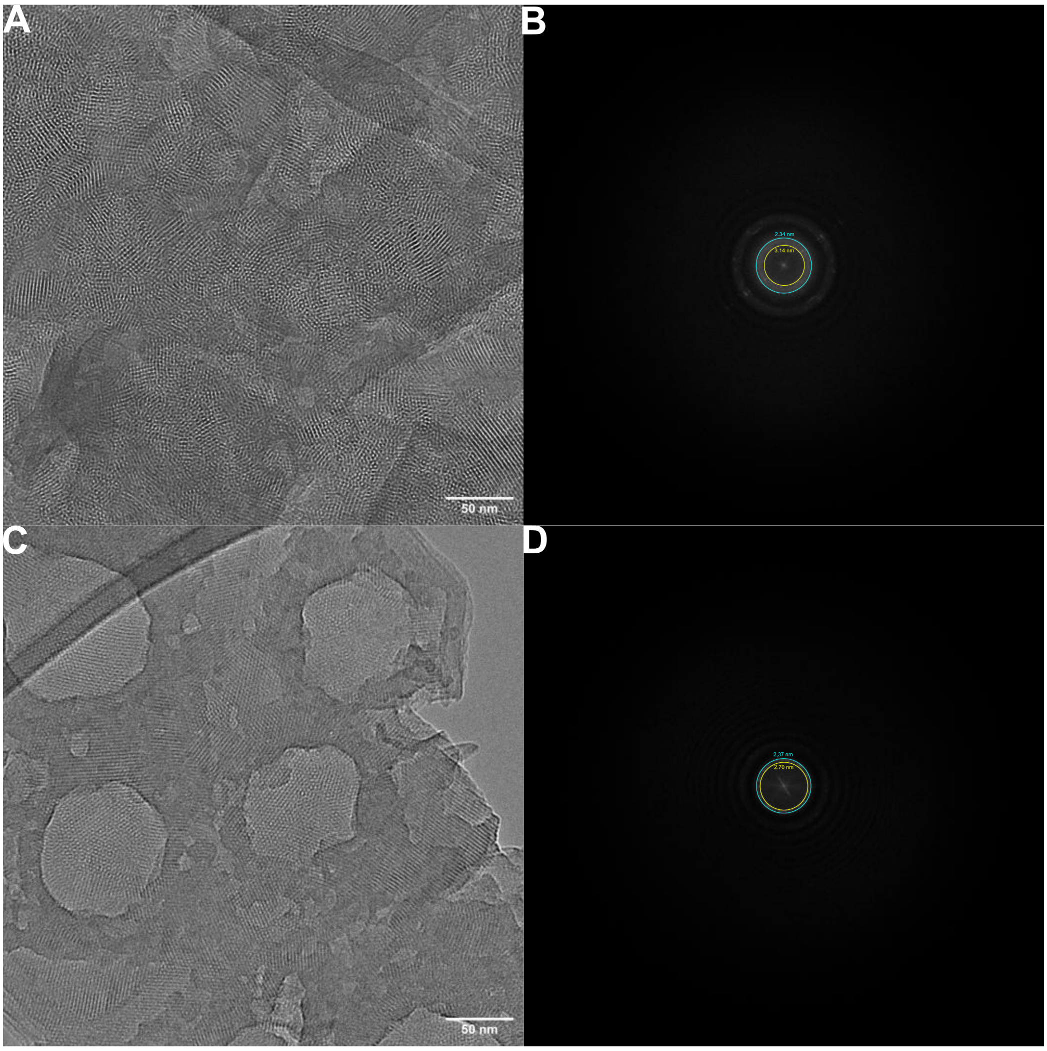

Figure S 21: TEM image of the "Intergrown Crystallites" phase $1_{Q(50\%)}$-8-$10_{T(50\%)}$-COF (A, left) and the corresponding FFT (B, right) over the whole image with marked "smudged" ring. TEM image of the "Hollow spheres" phase $1_{Q(50\%)}$-8-$10_{T(50\%)}$-COF (C, left) and the corresponding FFT (D, right) over the whole image with marked "concise" ring. CTF rings are ruled out based on radial integration, while the complete drying of the sample under high vacuum excludes ice rings.
